# Development of Novel Triazolo-Thiadiazoles from Heterogeneous “Green” Catalysis as Protein Tyrosine Phosphatase 1B Inhibitors

**DOI:** 10.1038/srep14195

**Published:** 2015-09-21

**Authors:** C. P. Baburajeev, Chakrabhavi Dhananjaya Mohan, Hanumappa Ananda, Shobith Rangappa, Julian E. Fuchs, Swamy Jagadish, Kodappully Sivaraman Siveen, Arunachalam Chinnathambi, Sulaiman Ali Alharbi, M. E. Zayed, Jingwen Zhang, Feng Li, Gautam Sethi, Kesturu S. Girish, Andreas Bender, Basappa, Kanchugarakoppal S. Rangappa

**Affiliations:** 1 Laboratory of Chemical Biology, Department of Chemistry, Bangalore University, Palace Road, Bangalore 560001, India; 2 Department of Studies in Chemistry, University of Mysore, Manasagangotri, Mysore-570006, India; 3 Frontier Research Center for Post-genome Science and Technology, Hokkaido University, Sapporo 0600808, Japan; 4 Centre for Molecular Informatics, Department of Chemistry, University of Cambridge, Lensfield Road, CB2 1EW, Cambridge, United Kingdom; 5 Department of Pharmacology, Yong Loo Lin School of Medicine, National University of Singapore-117597, Singapore; 6 Department of Botany and Microbiology, College of Science, King Saudi University, Riyadh -11451, Kingdom of Saudi Arabia; 7 Department of Studies in Biochemistry, University of Mysore, Manasagangotri, Mysore-570006, India

## Abstract

Condensed-bicyclic triazolo-thiadiazoles were synthesized *via* an efficient “green” catalyst strategy and identified as effective inhibitors of PTP1B *in vitro*. The lead compound, 6-(2-benzylphenyl)-3-phenyl-[1,2,4]triazolo[3][1,3,4]thiadiazole (BPTT) was most effective against human hepatoma cells, inhibits cell invasion, and decreases neovasculature in HUVEC and also tumor volume in EAT mouse models. This report describes an experimentally unidentified class of condensed-bicyclic triazolo-thiadiazoles targeting PTP1B and its analogs could be the therapeutic drug-seeds.

Protein tyrosine phosphorylation plays a pivotal role in various growth factors signaling to induce cell proliferation, differentiation, and survival^1^. Protein tyrosine kinases (PTKs) and protein tyrosine phosphatases (PTPs) are the two counteracting proteins which regulate tyrosine phosphorylation. PTKs phosphorylate specific tyrosine residues in their substrate and PTPs dephosphorylate them^2^,^3^. Dysregulation in the activities of PTKs and PTPs have been entangled with tumorigenesis. Recent findings suggest the involvement of PTPs in potentiating the action of PTKs resulting in the neoplastic transformation^4^. PTP1B is an ubiquitously expressed non-transmembrane phosphatase which belongs to the PTP superfamily and the implication of PTP1B in the dephosphorylation of Src (Y530) is well-demonstrated in progression of oncogenesis in breast cancer cells^5^. More recently, involvement of PTP1B in regulation of multiple signaling cascades to promote tumor progression and survival is elucidated in various cancers^6^,^7^,^8^. Several PTP1B small molecule inhibitors have been designed and proved to induce their effect at sub-micro molar concentration but failed to possess increased bioavailability due to reduced cell permeability and hydrophobicity^9^. Therefore, PTP1B has been emerged as a promising next generation therapeutic target to design novel, effective, easily bioavailable drugs to fight against cancer^10^. Herein, we adopted the synthetic strategy that could generate libraries of biologically active condensed-bicyclic triazolo-thiadiazoles (CBTT), which are identified as inhibitors of PTP1B via structure-based virtual screening of over 2 million compounds of diverse scaffold in order make them orally available drugs with desirable physicochemical properties.

## Results

### Chemistry

**Figure 1:**
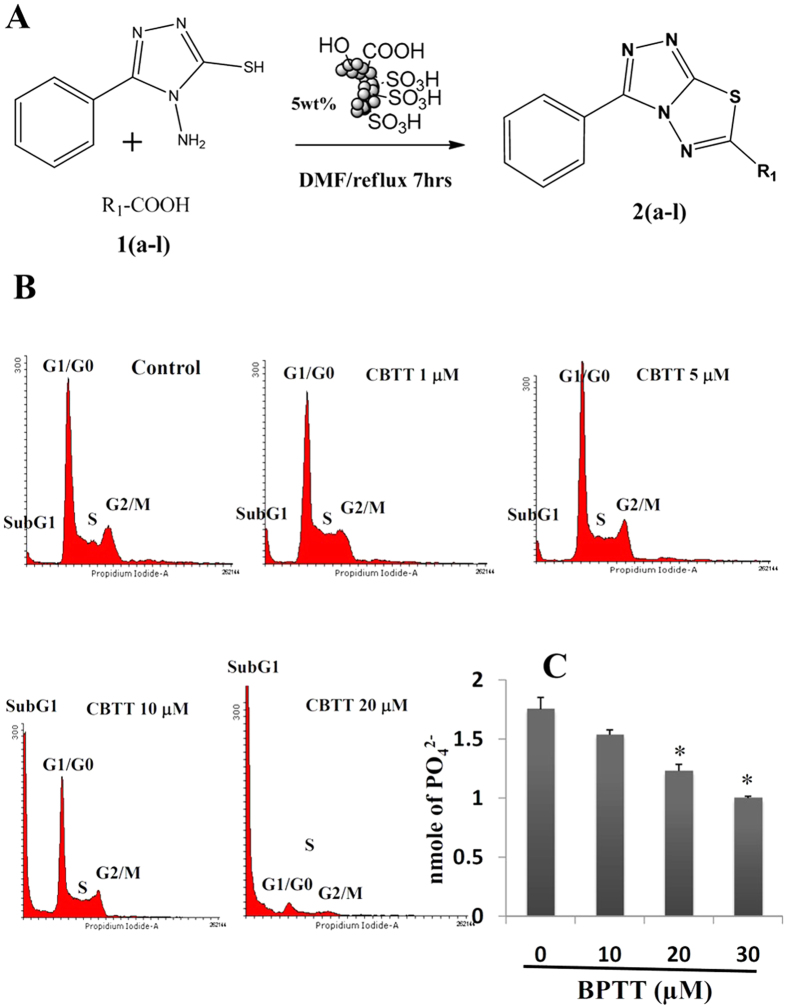
(**A**) Schematic representation for the synthesis of CBTTs; (**B**) BPTT causes arrest of HepG2 cells in SubG1 phase. Hepatoma cells treated with BPTT (0–20 μM) displayed the arrest of HepG2 cells in SubG1 phase of cell cycle. (**C**) Inhibition of the catalytic activity of PTP1B by BPTT was shown. PTP1B activity was measured by determining the free phosphate released from the PTP1B substrate with BPTT. Optical density was read at 620 nm and readings were converted to nmol of PO_4_^2−^. Each column represents the means of two determinations. *p < 0.05.

The synthesis of CBTT libraries entails the union of two nuclei, which accelerated the development of biologically interesting molecules in chemical biology and drug discovery. Various drugs including Triazolam, Ribavarin, Terconazole, Itraconazole, Etizolam, Fluconazole, Alprazolam, Furacylin and Cefazoline possess triazoles and thiadiazoles as the backbone and have been built on these heterocyclic nuclei. Presence of = N-C-S moiety could be the possible reason for the broad spectrum of pharmacological effects of triazole-thiadiazoles^11^,^12^. On the other hand, effective synthesis of CBTT scaffold requires highly hazardous phosphorous oxy chloride and overnight stirring at reflux temperature. We previously reported the synthesis of CBTT scaffold using phosphorous oxy chloride as cyclizing agent^13^. Initially, we carried out the microwave-assisted one pot synthesis of 4-amino-5-phenyl-4H-1,2,4-triazole-3-thiol using methyl benzoate, hydrazine hydrate and carbon disulphides. In the next step, we focused to synthesize CBTT libraries by using heterogeneous solid-acid catalysis, which becomes ideal in green chemistry due to its decreased reactor and plant correction problems, reusability, environmentally benign, saving of energy and more simple and convenient procedure^14^,^15^. Further, we employed the application of recyclable solid-acid catalyst, called glycerol-based carbon-SO_3_H to prepare the novel 3,6-disubstituted(1,2,4)-triazolo(3,4-b)(1,3,4) thiadiazole derivatives. Further, the glycerol-based sulfonic acid functionalized carbon catalyst system was used to synthesize the libraries of CBTTs under optimized reaction conditions (Fig. 1A). The effect of solvents for the synthesis of CBTT was also studied (Supplementary Table 1). The experiments were performed to study the recyclability of the catalyst system and showed that there was a significant reduction in the yield of the product after the third run (Supplementary Table 2). Newly prepared novel CBTTs are shown in Supplementary Table 3. We also proposed the plausible mechanism for the use of green-catalyst for the first time (Supplementary Scheme 1).

### Pharmacology

#### Identification of potent antiproliferative agent in CBTTs

In order to know the bioactivity of CBTTs, we investigated the cytotoxic effect of newly synthesized compounds on HepG2 cells as described previously^16^,^17^. Among the panel of new triazolo-thiadiazole based compounds, (6-(2-benzylphenyl)-3-phenyl-[1,2,4]triazolo[3,4–b][1,3,4]thiadiazole) (BPTT) which possess benzyl phenyl substitutions on the triazolo-thiadiazole ring was presented as the most active antiproliferative agent against hepatoma (HepG2) cells with an IC_50_ of 3 μM. We considered the lead compound BPTT for further studies.

### BPTT causes arrest of HepG2 cells in SubG1 phase

During apoptosis, activation of caspase activated DNAse (CAD) leads to disintegration of genomic DNA into oligomeric fragments. The cells with lesser DNA content leads to the formation of hypodiploid cells and in turn accumulation of cells in SubG1 phase^18^. We further investigated the effect of BPTT on cell cycle distribution of HepG2 cells as described previously^18^,^19^. Hepatoma cells were treated with different concentrations of BPTT (0–20 μM) and evaluated for pattern of cell cycle distribution using propidium iodide. We found the arrest of HepG2 cells in SubG1 phase in the dose dependent manner with maximum activity at 20 μM (Fig. 1B). This result suggests that BPTT was capable of inducing substantial apoptosis in HepG2 cells.

### BPTT inhibits the protein expression of PARP, Bcl-2, Survivin, Caspase-3 and Cyclin D1 in HepG2 cells

**Figure 2:**
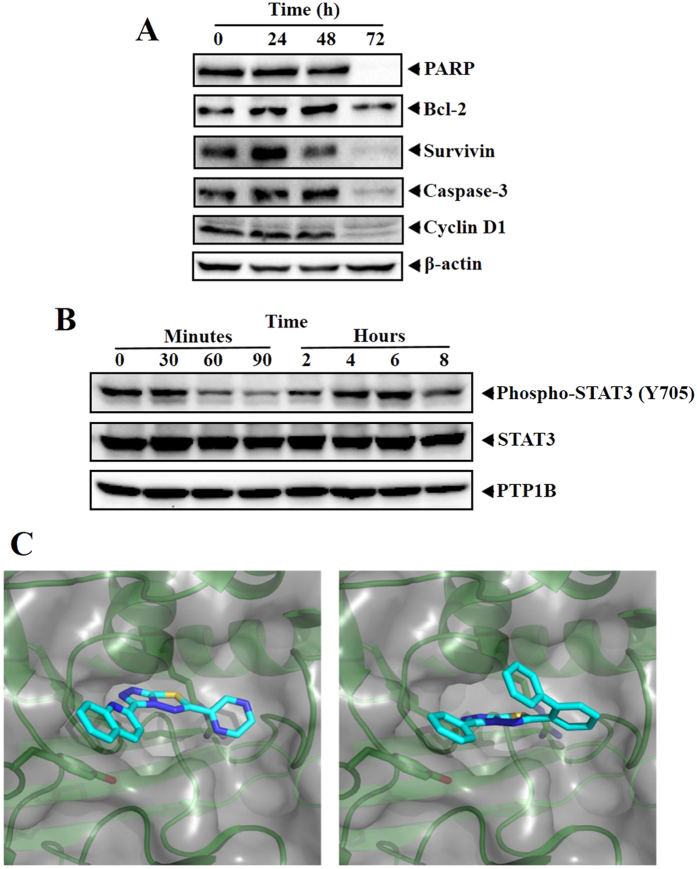
(**A**) BPTT inhibits the protein expression of PARP, Bcl-2, Survivin, Caspase-3 and Cyclin D1 in HepG2 cells. HepG2 cells were treated with BPTT, after which whole-cell extracts were prepared, and protein was resolved on SDS-PAGE gel, electrotransferred onto nitrocellulose membranes, and probed for interested antibodies and the gels in this figure are cropped, full length gels are presented in supplementary figure S1. (**B**) BPTT modulates the phosphorylation of STAT3 at tyrosine-705 in time dependent manner and has no effect on the protein expression of PTP1B and the gels in this figure are cropped, full length gels are presented in supplementary figure S1. (**C**) Molecular interaction studies of BPTT towards PTP1B; Predicted binding modes of a thiadiazole derivative of Park *et al.*^11^ (left) and BPTT (right) are highly similar.

Based on the observation that BPTT interferes with cell cycle distribution to induce its antiproliferative effect, we profiled the expression of cell cycle regulator (cyclin D1), apoptotic markers (caspase-3, and PARP) and antiapoptotic (Bcl-2, and Survivin) proteins. We observed the downregulation of aforesaid proteins in a time dependent manner and complete inhibition of these protein expression at 72 h (Fig. 2A).

### PTP1B inhibitory activity

Several studies have showed that CBTT’s are known to inhibit the catalytic activity of PTP1B. Among more than 2 million of diverse structures tested *in vitro*, CBTT was the only scaffold identified as the non-structural inhibitor for PTP1B^20^. Therefore, we considered the lead compound BPTT to evaluate for PTP1B inhibitory activity. We estimated the PTP1B enzyme activity by determining the free phosphate released from the PTP1B substrate in the presence and absence of BPTT using PTP1B inhibitor screening assay kit (*Abcam,* USA). The compound BPTT inhibited the catalytic activity of PTP1B by 45% and 38% at the concentration of 30 μM and 20 μM respectively (Fig. 1C). The inhibition of catalytic activity of PTP1B by BPTT was significantly higher compared to the previously reported structures^20^.

### BPTT modulates the phosphorylation of STAT3 in HepG2 cells

PTP1B have been reported to downregulate phosphorylation of STAT3 (Y705) and On the other hand, PTP1B inhibitors upregulate the phosphorylation of STAT3^21^. Based on this observation, we next evaluated the effect of BPTT on the phosphorylation of STAT3 at Y705. Western blotting analysis showed that treatment of HepG2 cells with BPTT initially resulted in a decreased STAT3 activation up to 60 minutes. Thereafter, a gradual increase in STAT3 phosphorylation was noted in a time dependent manner up to 8 h (Fig. 2B). On the other hand, PTP1B have also been demonstrated to interfere with VEGF-induced phosphorylation of VEGFR2 (Y1175)^22^. Hence, next, we examined the effect of BPTT on VEGF-stimulated phosphorylation of VEGFR2 in HUVEC. On treatment with BPTT, we observed only a marginal increase in the phosphorylation of VEGFR2 (data not shown).

### *In silico* interaction of BPTT with the phosphatase domain of the human PTP1B

Further, *in silico* docking was performed to rationalize and compare the molecular interactions of the newly synthesized CBTT libraries with the reported structures towards PTP1B. As Park *et al.* successfully employed computational techniques to study interactions of CBTT’s with PTP1B^20^ we aimed at a similar description of protein-ligand interactions based on an X-ray structure of the phosphatase domain of the human PTP1B (PDB: 2FH7). We prepared the structure for docking in MOE using protonate3D (Molecular operating environment) and removed two deeply buried water molecules resolved in the crystal structure to allow a binding mode similar to the predictions of Park *et al.* (waters 75 and 132). Computational docking studies predict the series of CBTTs to occupy the active site pocket of PTP1B similar to predictions of Park *et al* (Fig. 2C). The binding poses of CBTTs show major shape overlap and position aromatic rings in similar positions. The thiadiazole shows hydrogen bonding to the protein backbone whilst other fragments form cation-pi interactions with Arg-1595 and pi-pi interactions with Tyr-1422 respectively. In summary, we found that the newly synthesized compounds could serve as lead-structures that targets PTP1B.

### BPTT mitigates VEGF-induced HUVEC capillary-like structure formation and viability *in vitro*

**Figure 3:**
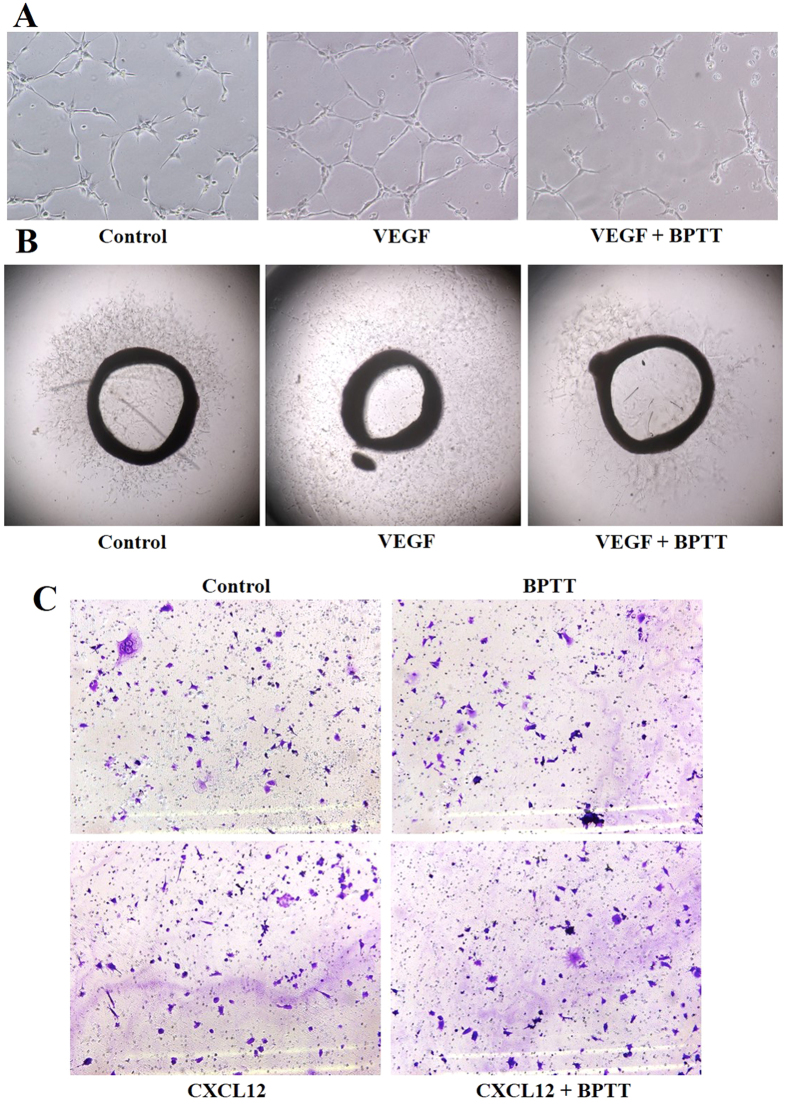
(**A**) *In vitro* anti-angiogenic activity of BPTT using HUVEC. In presence of VEGF, HUVECs form tubular structures on the Matrigel and in the presence of BPTT substantially decreased the continuity and number of HUVEC capillary-like structures. (**B**) Inhibitory activity of BPTT on rat-aortic ring formation by fibro-adipose tissue of Sprague-Dawley rats. The treatment of BPTT significantly inhibited VEGF-induced sprouting of microvessels. (**C**) *In vitro* anti-invasive activity of BPTT using HepG2 cells. In this assay system, we used CXCL12 as an inducer of invasion of HepG2 cells. The treatment with HepG2 cells reduced the motility of cells that could invade Matrigel. Data are the representatives of three independent experiments. *p < 0.05.

Sanggenon C is a phytochemical known to have inhibitory activity against PTP1B and angiogenesis^23^,^24^,^25^. Therefore, we evaluated the effect of BPTT on angiogenesis using *in vitro* capillary tube formation assay which represents a simple, reliable and powerful model for studying inhibitors of angiogenesis^26^. We examined the effect of BPTT on tubulogenesis in HUVECs in the presence and absence of VEGF as described previously^27^. When HUVECs were cultured on Matrigel, they spontaneously form three dimensional capillary-like tubular structures. In presence of VEGF, HUVECs form robust tubular-like structures when seeded on growth factor–reduced two-dimensional Matrigel and BPTT treatment substantially decreased the continuity and number of HUVEC capillary-like structures (Fig. 3A).

### BPTT suppresses VEGF-induced microvessel formation *ex vivo*

Further, the anti-angiogenic potential of BPTT was demonstrated using the rat thoracic aortic ring assay, an *ex vivo* angiogenesis model^28^. The serum-free three-dimensional rat aortic model closely resembles the complexities of *in vivo* angiogenesis from endothelial activation to pericyte acquisition and remodeling^26^. We observed the significant sprouting of microvessels on VEGF stimulation, leading to the formation of a network of vessels around the aortic rings. Treatment of BPTT significantly inhibited VEGF-induced sprouting of microvessels (Fig. 3B). The results of the capillary tube formation and rat aortic assays significantly support the multifaceted role of BPTT in antiangiogenesis.

### BPTT suppresses CXCL12 induced migration of HepG2 cells

PTP1B regulates the breast cancer cell invasion by modulating invadopodia dynamics^29^ and various studies have demonstrated the role of PTP1B in cancer cell invasion^30^. In order to determine the efficacy of BPTT against invasion of HepG2 cells, we performed *in vitro* invasion assay using Bio-Coat Matrigel invasion assay system (BD Biosciences, San Jose, CA, USA), as described earlier^31^. In this assay system, we used CXCL12 as an inducer and addition of CXCL12 was found to augment the invasive potential of HepG2 cells. On treatment with BPTT, we observed significant reduction in the motility of cells that could invade the Matrigel coated polycarbonate membrane, thereby indicating that BPTT substantially interferes with invasion of HepG2 cells (Fig. 3C).

### *In vivo* Ehrlich Ascites Tumor model

**Figure 4:**
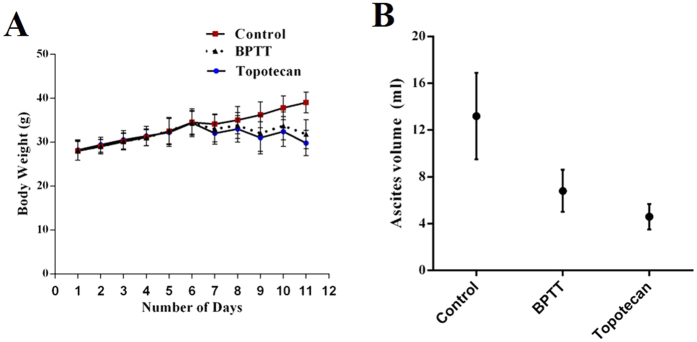
(**A**) Relative body weight of EAT bearing mice treated with vehicle alone, BPTT and topotecan. (**B**) Relative ascites fluid in EAT bearing mice treated with vehicle alone, BPTT and topotecan. Data are represented as mean ± S.E. *p < 0.05.

**Figure 5:**
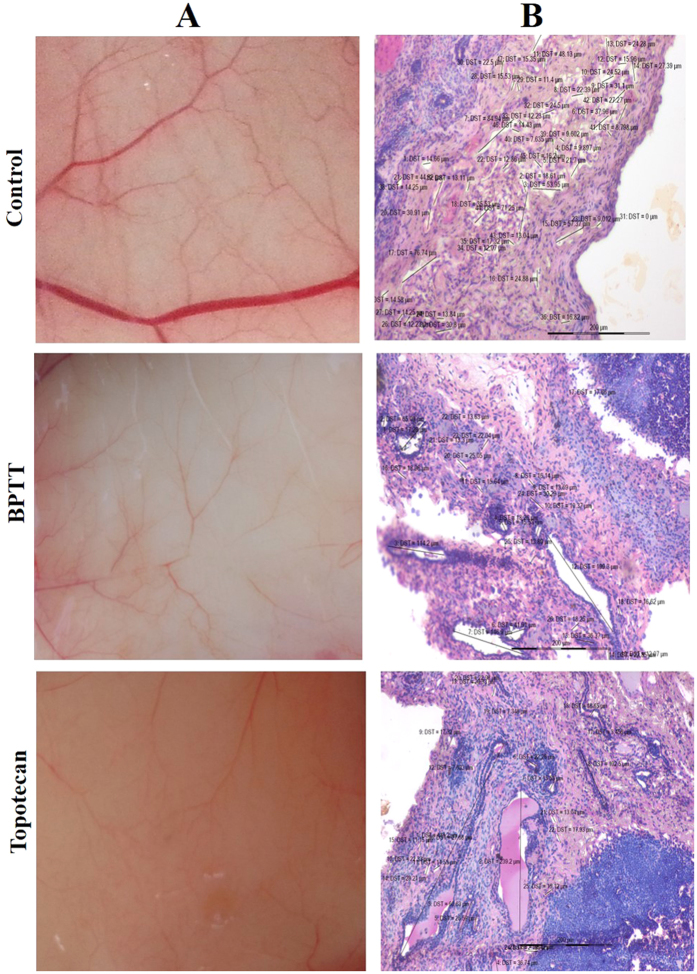
(**A**) *In vivo* anti-angiogenic activity of BPTT in the peritoneal cavity of EAT implanted mouse model. (**B**) Analysis of angiogenesis (MVD) in the peritoneum of EAT implanted mouse using H & E staining.

Given the relevance with the results of *ex vivo* experiments, we also evaluated the *in vivo* antiangiogenic potential of BPTT *via* intraperitoneal administration in an Ehrlich ascites tumor model as described earlier^32^,^33^. It was found that BPTT at the concentration of 10 mg/kg induced significant decrease of body weight, tumor volume (Fig. 4A,B) and peritoneal angiogenesis (Fig. 5A) compared with the DMSO-treated controls. The unpaired ANOVA *t* test showed a statistically significant difference in tumor growth between the BPTT-treated and control groups. We further measured microvessel density in peritoneum of experimental animals using H & E staining and found the reduction of angiogenesis by 44.68% and 46.81% in BPTT and topotecan treated group respectively (Fig. 5B).

## Discussion

In the present study, we report the novel, bioactive triazolo-thiadiazole derivative that targets PTP1B and demonstrated the mechanism of action *in vitro* and *in vivo*. PTP1B is an abundantly expressed protein tyrosine phosphatase associated with endoplasmic reticulum and it has been linked with regulation of several cellular events including cell growth, differentiation and transformation^5^. It was identified that PTP1B negatively regulates tyrosine phosphorylation of several upstream signaling proteins including c-Src, JAK2, VEGFR2 and insulin receptors^29^,^34^. Though SHP1, SHP2 and HCPTPA are known to interact with c-Src, JAK2 and VEGFR2, their role in physiological conditions remain unclear^35^,^36^,^37^. The main challenge in targeting PTP1B relies in selective inhibition due to high degree of homology among PTPs^9^. The prior idea about the CBTT being an effective scaffold against PTP1B, we identified and evaluated lead structure BPTT against PTP1B enzyme activity. Further, we validated the effect of BPTT on STAT3 and VEGFR2 in HepG2 cells and HUVECs respectively. Initially up to 90 mins, we observed the decrease in STAT3 phosphorylation, this is unexpected because PTP1B dephosphorylates JAK2 in turn downregulates STAT3 phosphorylation. However, we observed gradual increase in STAT3 phosphorylation (Y705) after 90 mins of incubation with BPTT. We did not observe change in the protein expression of PTP1B indicating that BPTT directly interferes with the catalytic activity and had no effect on its protein expression. PTP1B negatively regulates autophosphorylation of VEGFR2 *via* binding to the cytoplasmic domain of VEGFR2 in endothelial cells^35^. We tried to analyze phospho-VEGFR2 in BPTT treated HUVECs and observed only a marginal increase in the phosphorylation (data not shown). Sanggenon C, a phytochemical isolated from Chinese medicinal plant *Morus mongolica* has been found to exhibit inhibitory activity against PTP1B and also demonstrated to possess antiangiogenic properties *via* inhibition of hypoxia-inducible factor-1α. BPTT appears to have similar inhibitory effect against PTP1B and angiogenesis and needs to be explored further for its potential against human diseases.

## Conclusion

Herein, for the first time we report the use of heterogeneous “green” catalyst to prepare the novel triazolo-thiadiazoles. We have depicted target of lead structure as PTP1B and experimentally demonstrated the same. Moreover, this report describes an experimentally unidentified class of small molecules targeting PTP1B. Further derivatization of the lead structure may result in drug-seeds with enhanced PTP1B inhibitory activity. This finding opens an avenue for the development of novel triazolo-thiadiazoles based small molecules as therapeutic agents that target PTP1B in human diseases.

## Methods

### Synthesis

The synthesis and physical & chemical characterization, and spectral data on CBTT were provided as a supplementary file.

### MTT assay

The anti-proliferative effect of new compounds against HepG2 cells was determined by the MTT dye uptake method as described previously^38^. Briefly, the cells (2.5 × 10^4^/ml) were incubated in triplicate in a 96-well plate in the presence or absence of different concentrations of BPTT in a final volume of 0.2 ml for indicated time intervals at 37 °C. Thereafter, 20 μl MTT solution (5 mg/ml in PBS) was added to each well. After 2 h incubation at 37 °C, 0.1 ml lysis buffer (20% SDS, 50% dimethyl-formamide) was added; incubation was continued overnight at 37 °C; and then the optical density (OD) at 570 nm was measured by Tecan plate reader.

### Flow cytometric analysis

The effect of BPTT on cell cycle of HepG2 cells was performed as described previously^18^. To determine the effect of BPTT on the cell cycle, cells were treated with BPTT at indicated doses up to 48 h. Thereafter cells were washed, fixed with 70% ethanol, and incubated for 30 min at 37 °C with 0.1% RNase A in PBS. Cells were then washed again, resuspended, and stained in PBS containing 25 μg/ml propidium iodide (PI) for 30 min at room temperature. Cell distribution across the cell cycle was analyzed with a BD FACSVerse flow cytometer.

### Western Blotting

Western blot analysis was performed as previously described^39^. Briefly, BPTT treated MDA-MB-231whole-cell extracts were lysed in lysis buffer (20 mM Tris, pH 7.4), 250 mM NaCl, 2 mM EDTA (pH 8.0), 0.1% Triton X-100, 0.01 mg/ml aprotinin, 0.005 mg/ml leupeptin, 0.4 mM PMSF, and 4 mM NaVO_4_). Lysates were then spun at 14,000 rpm for 10 min to remove insoluble material and protein concentration was quantified. Thereafter, proteins were resolved on SDS gel. After electrophoresis, the proteins were electrotransferred to a nitrocellulose membrane, blocked with 5% nonfat milk, and probed with various antibodies overnight at 4 °C. The blot was washed, exposed to HRP-conjugated secondary antibodies for 1 h, and finally examined by chemiluminescence (ECL; GE Healthcare).

### PTP1B inhibitory activity

PTP1B activity was measured by determining the free phosphate released from the PTP1B substrate with a PTP1B Inhibitor Screening Assay kit from Abcam. 2xPTP1B substrate solutions (120 μM), 2xPTP1B Enzyme solutions (2 ng/well) and BPTT solutions (0–30 μM) were prepared and added to the 96 wells plate as described in the kit instructions. After incubations at 30 °C for 30 mins, reactions were then terminated by addition of 25 μl of Red Reagent and optical density is read at 620 nm. The readings were converted to nmol of PO_4_^2−^ with a phosphate standard curve.

### Molecular Modeling

*In silico* docking was carried out using MOE. The coordinates of the thiadiazole moiety, kindly provided by Park *et al.*, were chosen as grid centre for docking with default parameters in MOE. Predicted binding poses were visualized using Pymol.

### Invasion assay

The *in vitro* invasion assay was performed using Bio-Coat Matrigel invasion assay system (BD Biosciences, San Jose, CA, USA), according to the manufacturer’s instructions. 1 × 10^5^ HepG2 cells were suspended in serum-free DMEM and seeded into the Matrigel transwell chambers consisting of polycarbonate membranes with 8 μm pores. After pre-incubation with or without BPTT for 8 h, the transwell chambers were then placed into appropriate wells of a 24-well plate, in which either the basal medium only or basal medium containing CXCL12 had been added. After incubation, the upper surfaces of the Transwell chambers were wiped with cotton swabs, and the invading cells were fixed and stained with crystal violet solution. The invading cells were then counted in five randomly selected areas under microscopic observation.

### Capillary-like tube formation assay

Tube formation was assessed as described previously^27^. Briefly, HUVECs were pretreated with various dilutions of BPTT for 12 h and then seeded onto the Matrigel layer in 24-well plates at a density of 5 × 10^4^ cells in Medium 200 with or without VEGF. After 6 h, tubular structure of endothelial cells was photographed using an inverted microscope. Three independent experiments were performed.

### Rat aortic ring assay

Rat aortic ring assay was performed as described previously^28^. In brief, aortas isolated from Sprague-Dawley rats were cleaned of fibro adipose tissue and colateral vessels and cut into approximately 1 mm long rings. The aortic rings were randomized into Growth Factor Reduced Matrigel-coated wells and further sealed with a 100 μl overlay of Matrigel. Medium 200 containing with and without VEGF along with different dilutions of BPTT was added to the wells and incubated at 37 °C/5% CO_2_ for 6 days. At the end of incubation, the microvessel sprouting formed were fixed and photographed using a Nikon inverted microscope (magnification, 100X). Two independent experiments were performed.

### *In vivo* tumor model studies

6–8 weeks old female Swiss albino mice were kept in a 12 h light and 12 h dark cycle and fed with standard chow formula and reverse osmosis water and were acclimatized for 1 week and were used for establishing Ehrlich ascites tumor (EAT) model for further experiments as described earlier^40^. Animals were obtained and maintained in the animal house, Department of Studies in Zoology, Manasagangotri, Mysuru, India. The animal experiment was reviewed and approved by the Institutional Animal Ethical Committee, University of Mysore, Mysuru. Animal handling and experiments were carried out in accordance with the guidelines of the Committee for the Purpose of Control and Supervision of Experiments on Animals (CPCSEA). Experimental animals were injected with 5 × 10^6^ viable EAT cells intraperitoneally, and weight of the animals were recorded daily up to 11^th^ day to analyze the tumor growth. To investigate the *in vivo* efficacy of BPTT, we injected 10 mg/kg body weight of BPTT into the peritoneum of the EAT bearing mice daily from the 5^th^ day of transplantation. The animals were sacrificed on 12^th^ day and ascites fluid was collected by making an incision in the abdomen. The number of microvessels in a hotspot adjacent to tumor mass was analysed using H & E staining to determine the effect of BPTT on angiogenesis. The peritoneal cavity of DMSO-treated, topotecan treated and BPTT treated animals were photographed.

### Statistical analysis

The mean values are expressed ± S.E. for control and experimental samples and each experiment is repeated a minimum of two times. Statistical analysis was performed by one-way ANOVA. P < 0.05 was considered statistically significant (GraphPad Prism version 6.0, GraphPad Software).

## Additional Information

**How to cite this article**: Baburajeev, C. P. *et al.* Development of Novel Triazolo-Thiadiazoles from Heterogeneous "Green" Catalysis as Protein Tyrosine Phosphatase 1B Inhibitors. *Sci. Rep.* **5**, 14195; doi: 10.1038/srep14195 (2015).

## Supplementary Material

Supplementary Information
