# Supplementary material for: Development of Novel Triazolo-Thiadiazoles from Heterogeneous “Green” Catalysis as Protein Tyrosine Phosphatase 1B Inhibitors
Source: Sci Rep. 2015 Sep 21;5:14195. doi: 10.1038/srep14195 (PMC4585680; doi:10.1038/srep14195)

**Development of Novel Triazolo-Thiadiazoles from Heterogeneous “Green” Catalysis as Protein Tyrosine Phosphatase 1B Inhibitors**

C. P. Baburajeev, Chakrabhavi Dhananjaya Mohan, Hanumappa Ananda, Shobith Rangappa, Julian E. Fuchs, Swamy Jagadish, Kodappully Sivaraman Siveen, Arunachalam Chinnathambi, Sulaiman Ali Alharbi, ME Zayed, Jingwen Zhang, Feng Li, Gautam Sethi, K. S. Girish, Andreas Bender, Basappaa, Kanchugarakoppal S. Rangappa

**Supplementary data**

**Materials and methods**

All solvents used were of analytical grade and reagents used were purchased from Sigma-Aldrich. All IR spectra were obtained in KBr disc on a Shimadzu FT-IR 157 Spectrometer.1H and 13C NMR spectra were recorded on a Bruker WH-200 (400MZ) spectrometer in CDCl3 or DMSO-d6 as solvent, using TMS as an internal standard and chemical shifts are expressed as ppm. Mass spectra were determined on a Shimadzu LC-MS. Microwave irradiation was carried out in a Anton Paar mono mode microwave-300. The progress of the reaction was monitored by TLC pre-coated silica gel G plates.

**Supplementary Table 1**: Optimisation of solvent for the synthesis of 2a in the presence of catalyst under reflux

| Entry | Solvent | Time (h) | Yield |
| --- | --- | --- | --- |
| 1 | Ethanol | 7 | NR |
| 2 | Chloroform | 7 | NR |
| 2 | Toluene | 7 | 45% |
| 3 | Acetonitrile | 7 | 50% |
| 4 | DMF | 7 | 78% |
| 5 | Water | 7 | NR |

It has been found that the product was formed in good yield by using DMF as a solvent under reflux condition for 7h.

**Supplementary Table 2**: Evaluation of the reuse of glycerol-based SO_3_H –Carbon catalyst for the synthesis of **2a.**

| Runs | 1 | 2 | 3 | 4 |
| --- | --- | --- | --- | --- |
| Yield | 78% | 74% | 45% | 31% |

The experiments performed to study the recyclability of the catalyst system employing the reaction showed that there was a significant reduction in the yield of the product after third run.

**Supplementary Table 3**:- The physical parameters of the newly synthesized heterocyclic compounds.

| **Entry** | **Acid (1a-l)** | **Product (2a-l)** | **Yield (%)** |
| --- | --- | --- | --- |
| **2a** | **1a** |  | **78** |
| **2b** | **1b** |  | **79** |
| **2c** | **1c** |  | **77** |
| **2d** | **1d** |  | **81** |
| **2e** | **1e** |  | **75** |
| **2f** | **1f** |  | **76** |
| **2g** | **1g** |  | **80** |
| **2h** | **1h** |  | **78** |
| **2i** | **1i** |  | **81** |
| **2j** | **1j** |  | **72** |
| **2k** | **1k** |  | **80** |
| **2l** | **1l** |  | **81** |

**Supplementary Scheme 1: Schematic representation for the cyclization of CBTT:** The First step involves the protonation of acid followed by dehydration and simultaneous attack of nitrogen lone pair to the electron deficient acylium ion to form an intermediate. In the second step, the intermediate undergo neighboring group participation with nucleophilic Sulphur, which leads to the formation of C-S bond by the elimination of water molecule. Finally, deprotonation results in the formation of the title products.

*Microwave synthesis of 4-amino-5-phenyl-4h-1,2,4-triazole-3-thiol.* A mixture of methylbenzoate (1mmol), and hydrazine hydrate (1mmol) in 20ml ethanol was irradiated by MW at 700 Watt in a specially designed Teflon vessel containing lead acetate, until all the starting material was consumed (1-2min, as monitored by TLC). To the above mixture add (0.006 mmol) of KOH, CS_2_ (1mmol) and further irradiated at 700Watt for 1min. Finally add hydrazine hydrate (2mmol) drop wise to the above mixture and continued the irradiation at 700Watt until a white solid appeared at the bottom (2-3min). The lead acetate worked as a trap for H_2_S that was evolved during reaction. The solid obtained dissolved in water (15-20ml) and acidified with Con HCl. The separated solid was filtered, dried and recrystallized to obtain pure *4-amino-5-phenyl-4h-1,2,4-triazole-3-thiol.*  Yield 78%, m.p. 232—234 ℃;*IR (KBr ) ᵧ/cm^-1^ : 3310.07 (NH_2_ stretch), 3071.36( aromatic CH stretch),1472.38(tautomeric c=s). 1H NMR:(400MHZ,DMSO-d6).* δ: 7.6-7.5 (m, 2H, ArH), 7.34—7.2 (m, 3H, ArH), 5.14 (s, 2H, NH_2_);

*General procedure for the synthesis of 6-substituted-3-phenyl-(1,2,4)-triazolo(3,4-b)(1,3,4-thiadiazole* ***(2a-2l)*** *by using glycerol-based SO_3_H-Carbon catalyst****.*** To a mixture of *4-amino-5-phenyl-4h-1,2,4-triazole-3-thiol* (1 mmol) and **(1a-l)**(1mmol) in DMF (5 ml) in a rbf added glycerol-based SO_3_H –Carbon catalyst(5wt% of 2). The reaction mixture was refluxed for 7h Completion of the reaction was monitored by TLC. Filter off the catalyst and wash with water. Remove the solvent under reduced pressure. To the concentrated mass add crushed ice. Reaction mixture P^H^ was adjusted to 8 by using K_2_CO_3_ and KOH. The solid separated out was filtered and washed with excess water. Dried and recrystalized from appropriate solvent.

***2a.****6-(benzo[b]thiophen-3-yl)-3-phenyl- [1,2,4]triazolo[3,4-b][1,3,4]thiadiazole.* IR(KBr)*v*/cm^-1^:3059.20(ArC-Hstr),1604.75(C=Nstr),1467.88(C=Cstr). ^1^HNMR(400MHz,DMSO-d6): *δ*:8.85 (s, 1H,Ar-H), 8.6 (d, 1H,Ar-H), 8.3(d,1H,Ar-H),8.15 (d,1H,Ar-H),7.7-7.5(m,6H,Ar-H);^13^CNMR(DMSO-*d*6)*δ*:167.81, 164.18,159.42,151.99,146.87,148.9,137.47,132.28,130.26,125.66,123.81, 121.01,120.89,116.19,;LCMS(MM:ES+APCI) 336.0(M+H)^+^

***2b.***3-chloro-6-(1-(3-phenyl-*[1,2,4]triazolo[3,4-b][1,3,4]thiadiazol-6-yl)ethyl)-9H-carbazole****.*** IR (KBr)*v*/cm^-1^: 3426.99 (N-Hstr), 3035.68 (ArC-Hstr), 2966 (methylC-Hstr), 1581.45 (C=Nstr), 1471.80 (C=Cstr). ^1^HNMR (400 MHz, DMSO-d6): ^1^HNMR (400 MHz, DMSO-d6) *δ*: 11.4 (s, 1H,N-H), 8.2 (m, 2H,Ar-H), 8.1-8.0 (m,2H,Ar-H),.7.7-7.6(m, 4H,Ar-H), 7.5(d, 1H,Ar-H), 7.3(d,1H,Ar-H),7.2(d,1H,Ar-H)4.8(q,1H,-CH),1.7(m,3H,-CH_3)_ : LCMS (MM:ES+APCI) 430.2(M+H)^+^

***2c.***6-(3,5-dimethyl-1H-pyrazol-4-yl)-3-phenyl-*[1,2,4]triazolo[3,4-b][1,3,4]thiadiazole****.*** IR(KBr)*v*/cm^-1^:3360.11(N-Hstr),3090.68(ArC-Hstr),1595.89(C=Nstr), 1471.74(C=Cstr). ^1^HNMR(400MHz,DMSO-d6):12.1(s,1H,-NH), 8.3(s,1H,Ar-H),8.1(s,1H,Ar-H),8.0-7.8(m,2H,Ar-H)7.6(m,1H,Ar-H),2.8(s,6H,=CH). *δ*: LCMS (MM:ES+APCI) 297.2(M+H)^+^

***2d***.1-methyl-3-(3-phenyl-[1,2,4]triazolo*[3,4-b][1,3,4]thiadiazol-6-yl)-1H-indazole.* IR(KBr)*v*/cm^-1^: 3064.12 (Ar-Hstr), 2937.11 (methylC-Hstr), 1599.45 (C=Nstr), 464.29 (C=Cstr). ^1^HNMR(400MHz,DMSO-d6):*δ*: 8.4 (d,1H,Ar-H),8.1(m,3H,Ar-H),8.0(m,1H,Ar-H),7.9(m,1H,Ar-H),7.7-7.6(m,1H,Ar-H), 7.3-7.1 (m,2H,Ar-H), 3.6(s,3H,-CH_3_); ^13^C NMR (DMSO-*d*6) *δ*: 168.2, 164.19,159.73,152.00, 147.45,132.26,127.27,126.64,123.40,121.70,118.93,114.48,113.62,110.66,45.38.; LCMS (MM:ES+APCI) 333.2(M+H)^+^

***2e.7***.6-(cylohexylmethyl)-3-phenyl-*[1,2,4]triazolo[3,4-b][1,3,4]thiadiazole*. IR (KBr) *v*/cm^-1^: 3035.68 (ArC-Hstr), 2966.38 (methylC-Hstr), 1605.45 (C=Nstr), 1469.81 (C=Cstr). ^1^HNMR (400MHz, DMSO-d6): 8.2 (d,2H,Ar-H),7.7-7.5(m,3H,Ar-H),2.9(d,2H,CH_2_),1.9-0.8(m,10H,Cyclohexyl),0.78(s,9H,-CH_3_);13CNMR (DMSO-*d*6) *δ*: 167.91, 164.82, 159.48,152.99,146.87,137.46,132.28,130.26,53.85, 46.13, 43.72, 28.63, 25.02, 21.13.; LCMS (MM:ES+APCI) 355.2(M+H)^+^

**2f.**6-(6-chloropyridin-2-yl)-3-phenyl-*[1,2,4]triazolo[3,4-b][1,3,4]thiadiazole***.**

IR(KBr)*v*/cm^-1^:3023.21(ArC-H),1604.83(C=Nstr),1469.81(C=Cstr),723.33(C-Clstr). ^1^HNMR(400MHz,DMSO-d6):*δ*:8.9(d,1H,Ar-H),8.8(d,1H,Ar-H), 8.2-8.3(m,2H,Ar-H),7.8-7.7(m,1H,Ar-H),7.5-7.6(m,3H,Ar-H):LCMS (MM:ES+APCI) 314.02(M+H)^+^

**2g.**(4-(3-phenyl-[*1,2,4]triazolo[3,4-b][1,3,4]thiadiazol-6-yl)pyridine-2-yl)methanamine***.**

IR(KBr)*v*/cm^-1^:3420.68(N-Hstr),3090.68(ArC-Hstr),1608.45(C=Nstr), 1471.44(C=Cstr). ^1^HNMR(400MHz,DMSO-d6): *δ*: 8.4 (d,1H,Ar-H), 8.2 (d,1H,Ar-H),7.7-7.5(m,3H,Ar-H),7.0-6.9(m,3H,Ar-H),4.6(m,2H,-CH_2_),2.8(s,2H,-NH_2_);13CNMR(DMSO-*d*6)*δ:168.27,165.94,153.47,147.94, 145.12,136.38,134.36,131.13,128.78,126.02,125.58,124.84,119.40,109.63,72.33* ;LCMS (MM:ES+APCI) 309.0(M+H)^+^

**2h.**6-(1-methyl-1H-pyrrol-2-yl)-3-phenyl-*[1,2,4]triazolo[3,4-b][1,3,4]thiadiazole***.**

IR(KBr)*v*/cm^-1^:3029.20(ArC-Hstr),2902.25(methylC-Hstr),1606.76(C=Nstr), 1469.54(C=Cstr). ^1^HNMR(400MHz,DMSO-d6)*δ*: 8.4 (d,1H,Ar-H), 8.0 (d,1H,Ar-H),7.9(m,1H,Ar-H),7.8(d,1H,Ar-H)7.7-7.6(m,1H,Ar-H),6.9(s,1H,Pyrole-CH),6.1-6.00(m,2H,Pyrole-CH), 3.5(s, 3H,-CH_3_); 13C NMR (DMSO-*d*6) *δ*: 168.2, 161.9, 159.9,155.6,145.4,136.5,122.9, 120.2, 116.6, 112.5,37.2 ; LCMS (MM:ES+APCI) 282.0(M+H)^+^

**2i.**6-(benzo[d][1,3]dioxol-5-yl)-3-phenyl-*[1,2,4]triazolo[3,4-b][1,3,4]thiadiazole***.**

IR(KBr)*v*/cm^-1^:3030.55(ArC-Hstr),1585.43(C=Nstr),1471.88(C=Cstr). ^1^HNMR(400MHz,DMSO-d6):*δ*:8.3(d,2H,Ar-H),7.6-7.4(m,5H,Ar-H),7.1(d,1H,Ar-H),6.1(s,2H,-CH_2_),LCMS(MM:ES+APCI)323.0(M+H)^+^.

**2j.**3-(trifluoromethyl)-2-methyl-N-(2-(3-phenyl-*[1,2,4]triazolo[3,4-b][1,3,4]thiadiazol-6-yl)benzenamine***.**

IR(KBr)*v*/cm^-1^:3090.68(ArC-Hstr),1605.45(C=Nstr),1467.88(C=C). ^1^HNMR(400MHz,DMSO-d6):*δ*:9(s1H,Ar-H),8.3(d,1H,Ar-H),8.2(m,2H,Ar-H),8.1(d,1H,Ar-H),7.8(dd,1,H,Ar-H),7.5(m,3H,Ar-H),7.4(d,2H,Ar-H),7.3(m,1H,Ar-H),7.0(m,1H,Ar-H),2.2(s,3H,-CH_3_).; LCMS (MM:ES+APCI) 452.0(M+H)^+^

**2k.** 6-(2-benzylphenyl)-3-phenyl-*[1,2,4]triazolo[3,4-b][1,3,4]thiadiazole*.

IR(KBr)*v*/cm^-1^:3034.78 (ArC-Hstr), 2858.18(methylC-Hstr),1599.45(C=Nstr), 1467.88(C=Cstr).^1^HNMR(400MHz,DMSO-d6): *δ*: 8.0(d,2H,Ar-H), 7.7(d,1H,Ar-H), 7.6(m,1H,Ar-H),7.5-7.3(m, 6H,Ar-H), 7.2(m, 2H,Ar-H),7.0(d,2H,Ar-H),4.3(s,2H,-CH_2_) ; 13C NMR (DMSO-*d*6) *δ*: 168.89, 164.19, 159.73,152.02, 147.45, 142.43, 138.44,137.46,132.26,127.27,125.64,123.40,121.70,55.23,;LCMS (MM:ES+APCI) 369.3(M+H).^+^

**2l.**3-(3-phenyl-*[1,2,4]triazolo[3,4-b][1,3,4]thiadiazol-6yl)-1-p-tolylpropan-1-one.*

IR IR(KBr)*v*/cm^-1^:3068.85(ArC-Hstr),2931.90(methyC-Hstr),1662.69(=COstr) 1608.69(C=Nstr),1462.09(C=Cstr). ^1^HNMR(400MHz,DMSO-d6):*δ*:8.3 (m,1H,Ar-H),8.2(m,1H,Ar-H),8.1(m,2H,Ar-H),7.9-7.7(m,3H,Ar-H),7.5(m,2H,Ar-H), 3.1(m,2H,-CH_2_),2.8(m,2H,-CH_2_),2.3(s,3H,-CH_3_);13CNMR(DMSO-*d*6)*δ*: 179.35,167.81,164.13, 158.14,152.17,149.20,138.81,135.10,132.39,130.13,127.60,125.99,124.24,36.13,29.03,22.00.; LCMS (MM:ES+APCI) 349.2(M+H)^+^

***(2a)***

6-(benzo[b]thiophen-3-yl)-3-phenyl- [1,2,4]triazolo[3,4-b][1,3,4]thiadiazole

IR(KBr)*v*/cm^-1^:3059.20(ArC-Hstr),1604.75(C=Nstr),1467.88(C=Cstr). ^1^HNMR(400MHz,DMSO-d6): *δ*:8.85 (s, 1H,Ar-H), 8.6 (d, 1H,Ar-H), 8.3(d,1H,Ar-H),8.15 (d, 1H,Ar-H), 7.7-7.5 (m, 6H,Ar-H), ; ^13^C NMR (DMSO-*d*6) *δ*: 167.81, 164.18,159.42,151.99,146.87,148.9,137.47,132.28,130.26,125.66,123.81, 121.01,120.89,116.19,;LCMS(MM:ES+APCI) 336.0(M+H)^+^


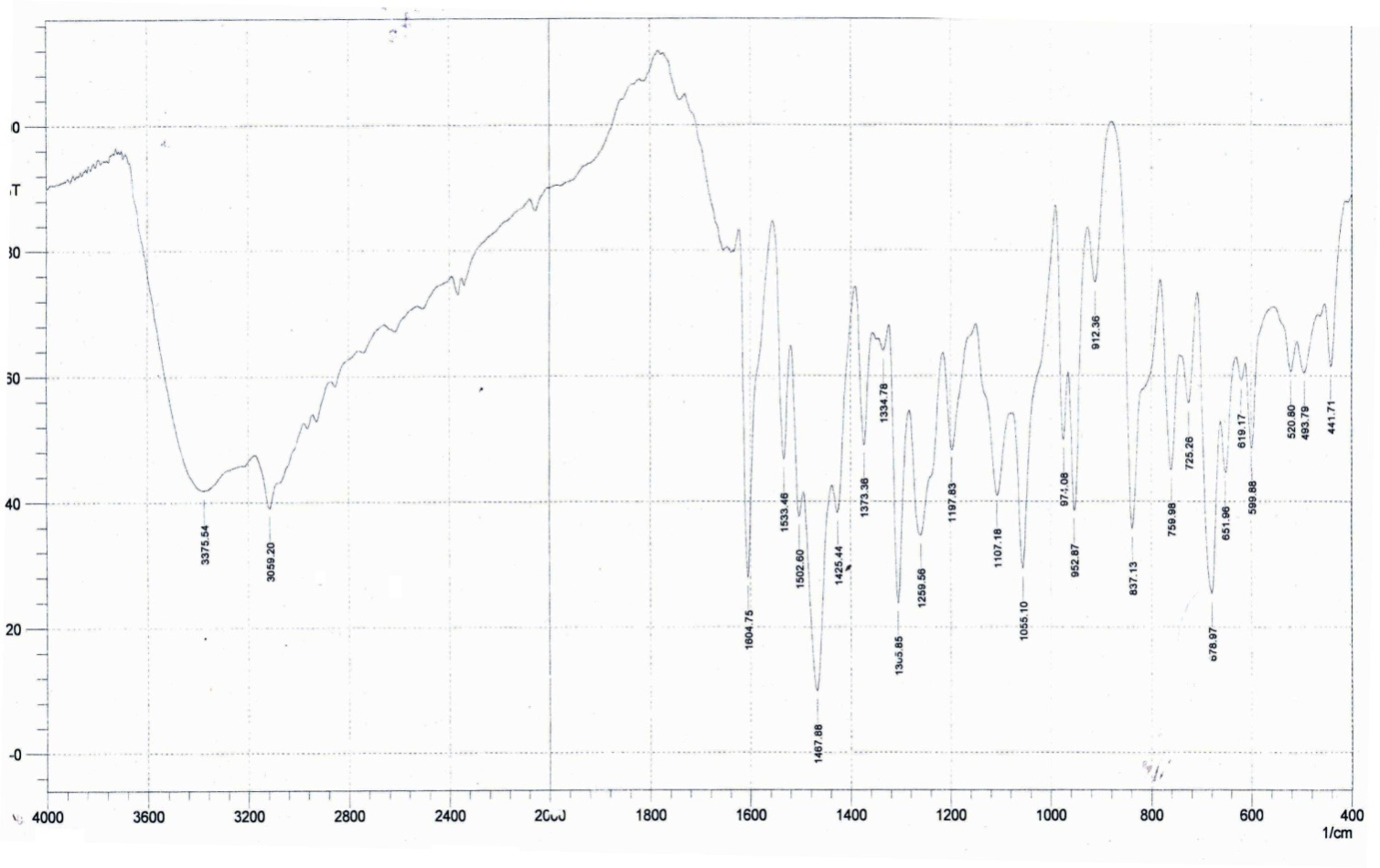

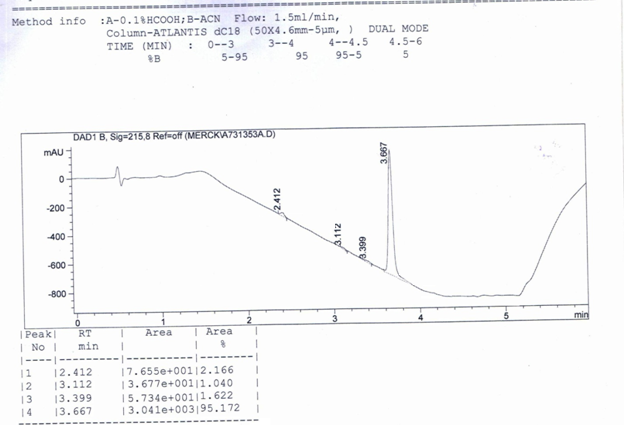


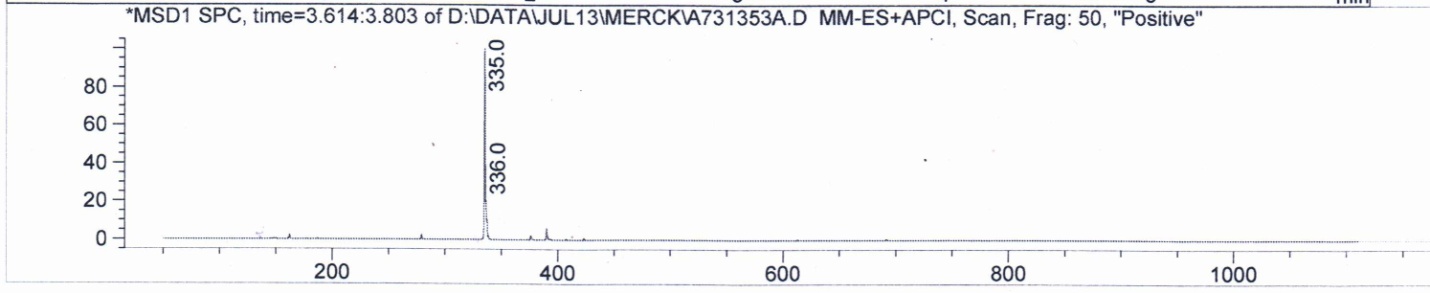


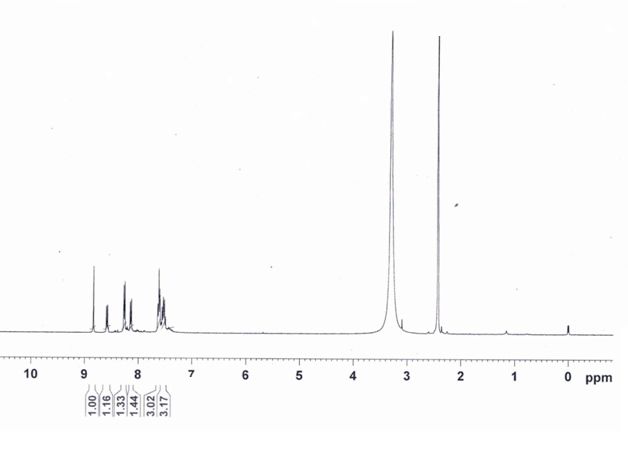


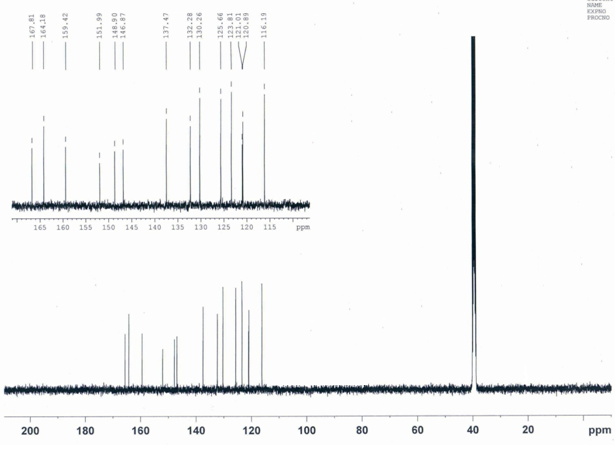


***(2b)***

3-chloro-6-(1-(3-phenyl-*[1,2,4]triazolo[3,4-b][1,3,4]thiadiazol-6-yl)ethyl)-9H-carbazole*

IR(KBr)*v*/cm^-1^:3426.99(N-Hstr),3035.68(ArC-Hstr),2966(methylC-Hstr),1581.45(C=Nstr),1471.80(C=Cstr).^1^HNMR(400MHz,DMSO-d6): ^1^HNMR(400MHz,DMSO-d6): *δ*: 11.4 (s, 1H,N-H), 8.2 (m, 2H,Ar-H), 8.1-8.0 (m,2H,Ar-H),.7.7-7.6(m, 4H,Ar-H), 7.5(d, 1H,Ar-H), 7.3(d,1H,Ar-H),7.2(d,1H,Ar-H)4.8(q,1H,-CH),1.7(m,3H,-CH_3)_ : LCMS (MM:ES+APCI) 430.2(M+H)^+^


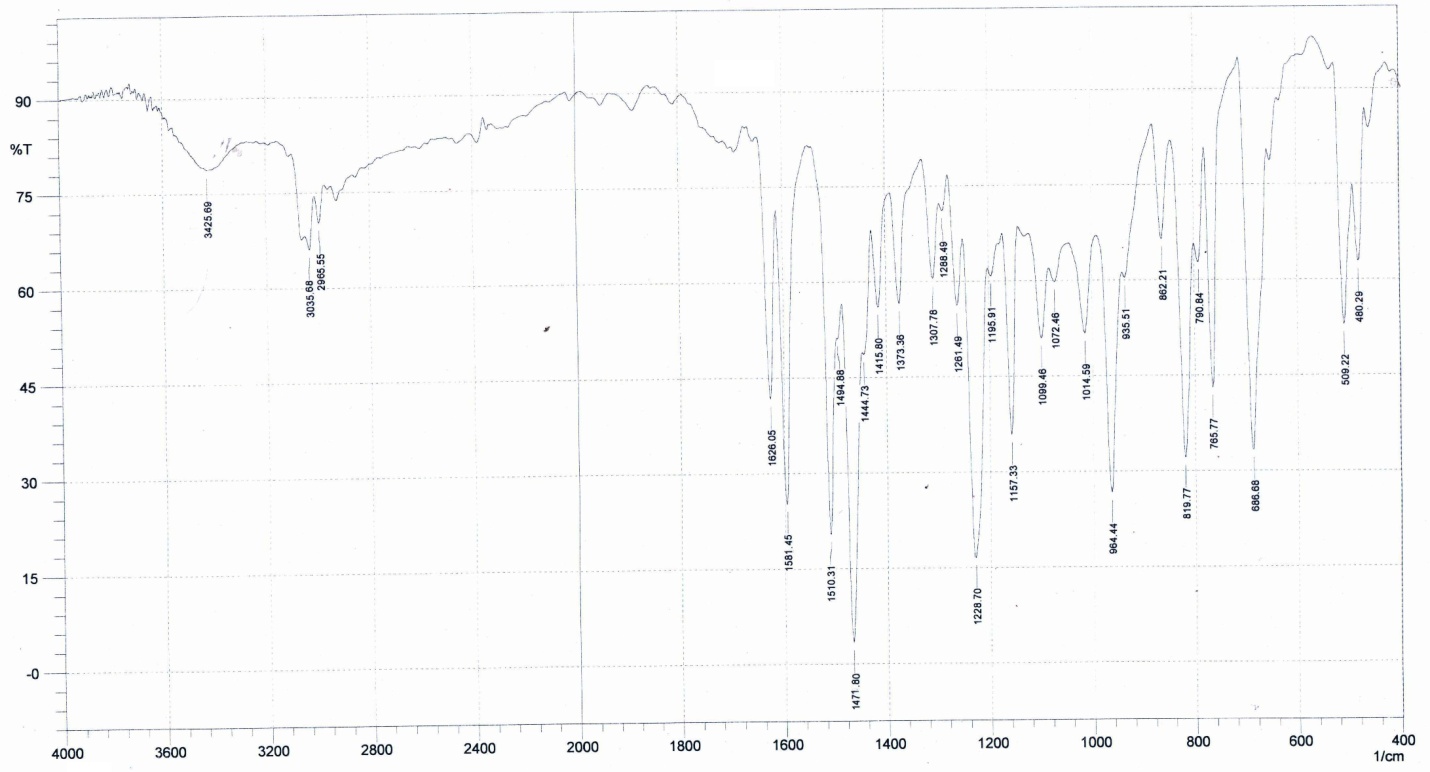


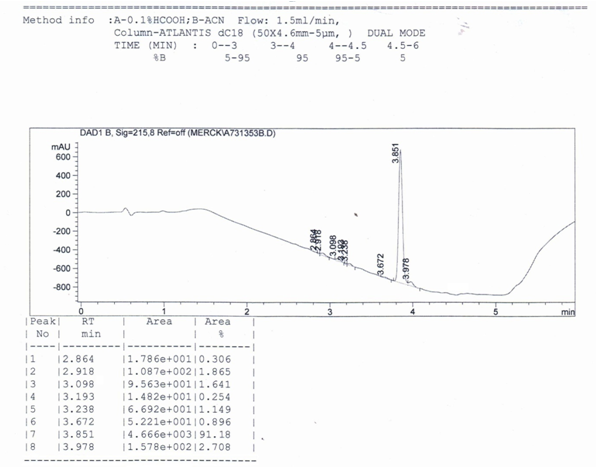


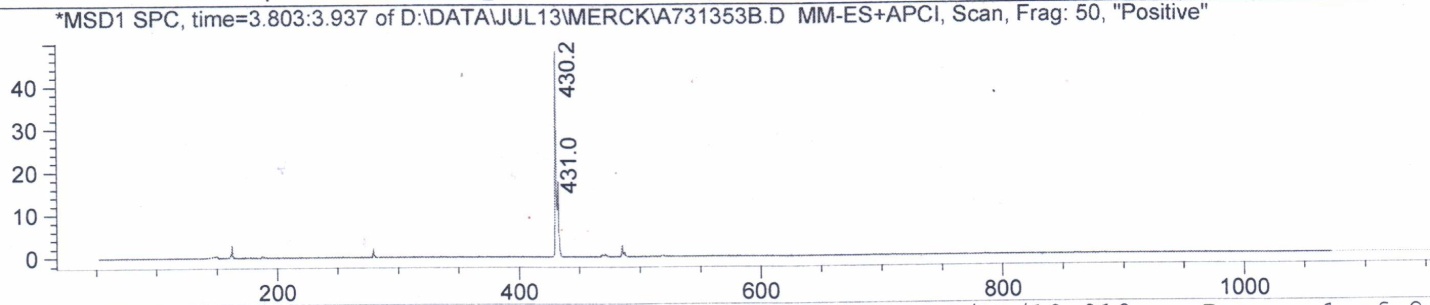


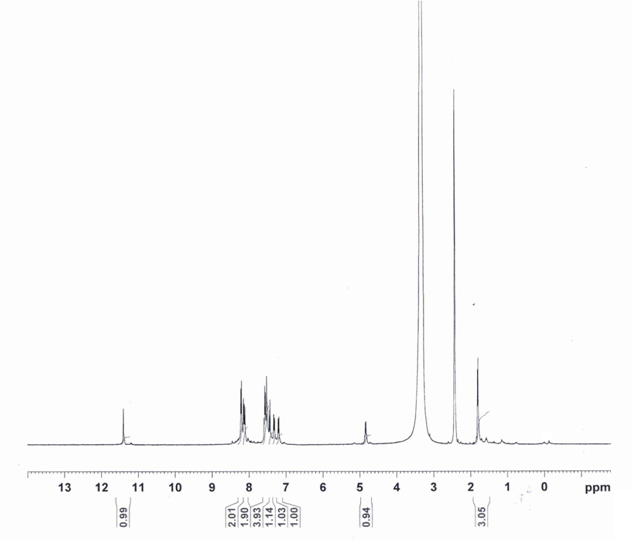


***(2c)***

6-(3,5-dimethyl-1H-pyrazol-4-yl)-3-phenyl-*[1,2,4]triazolo[3,4-b][1,3,4]thiadiazole*

IR(KBr)*v*/cm^-1^:3360.11(N-Hstr),3090.68(ArC-Hstr),1595.89(C=Nstr), 1471.74(C=Cstr). ^1^HNMR(400MHz,DMSO-d6):12.1(s,1H,-NH), 8.3(s,1H,Ar-H),8.1(s,1H,Ar-H),8.0-7.8(m,2H,Ar-H)7.6(m,1H,Ar-H),2.8(s,6H,=CH). *δ*: LCMS (MM:ES+APCI) 297.2(M+H)^+^


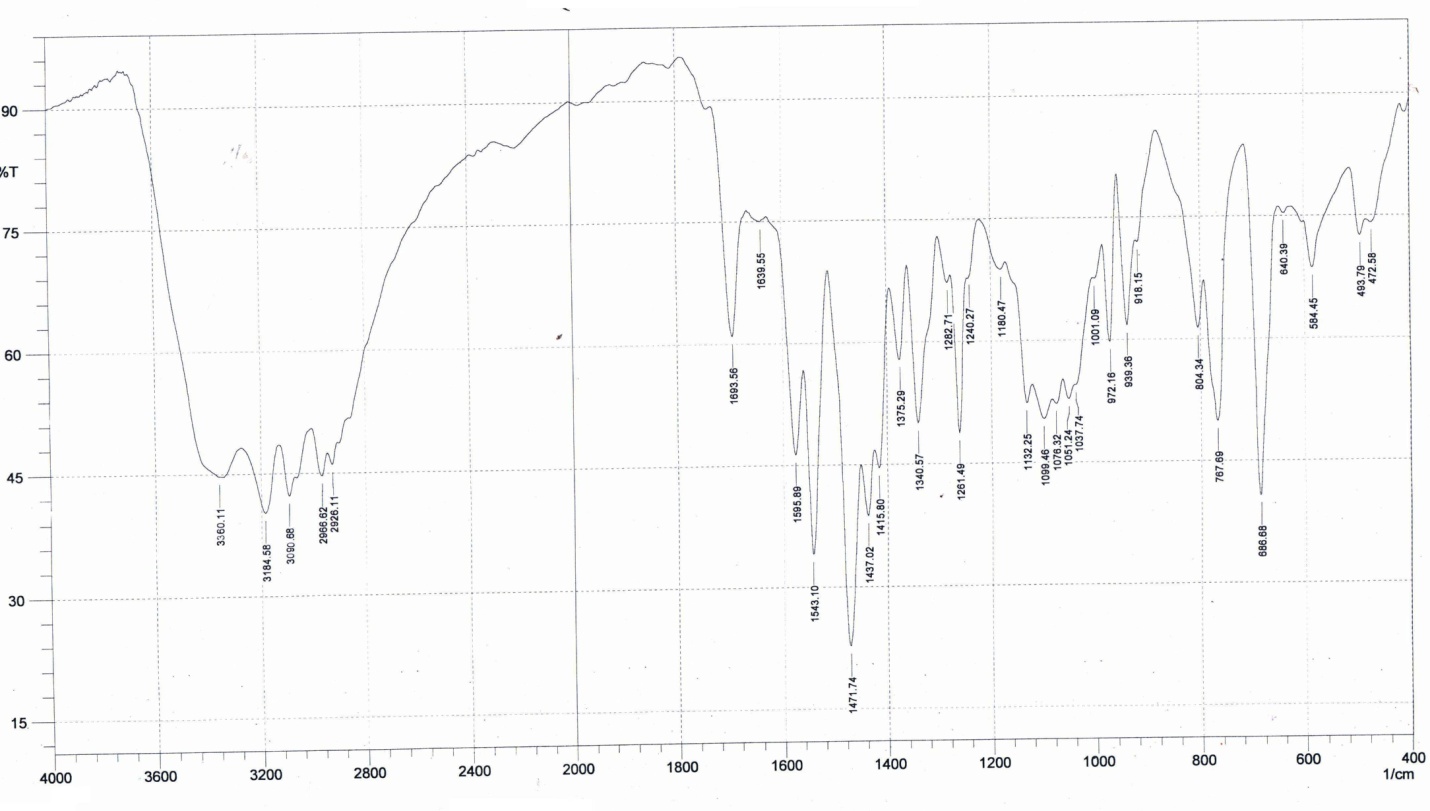


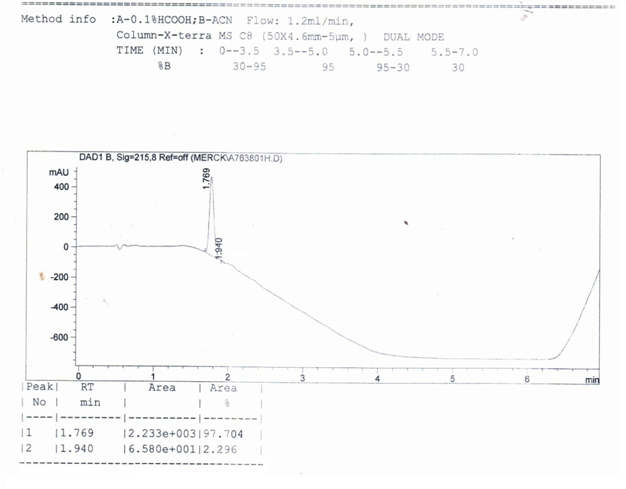


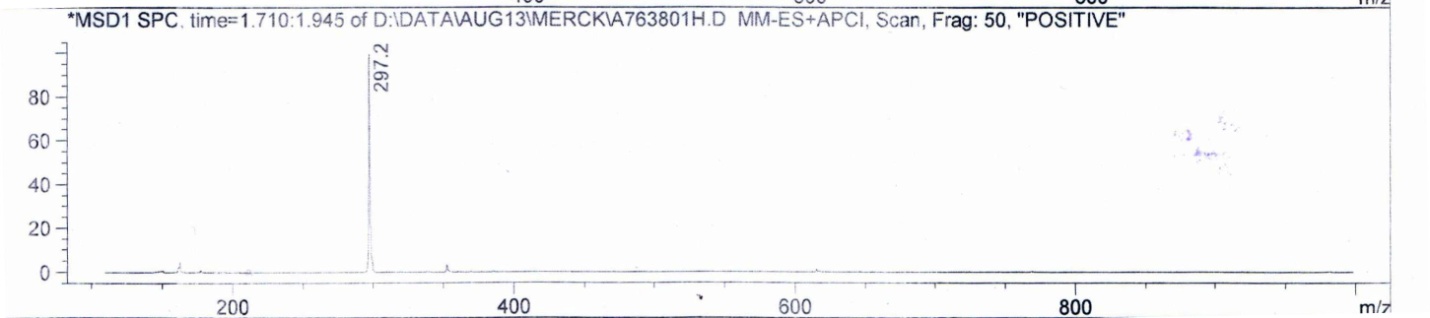


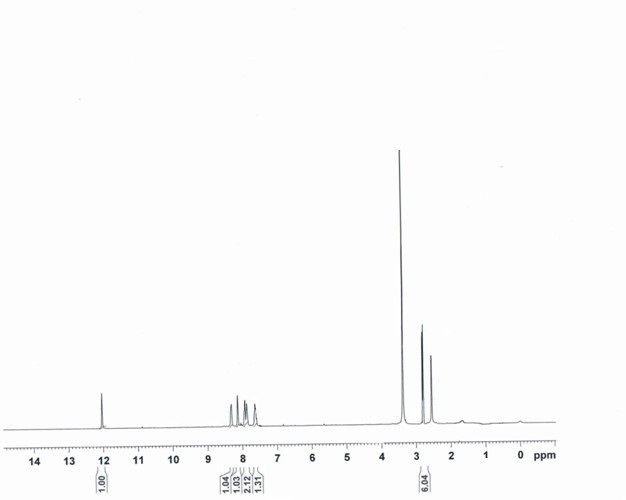


***(2d)***

1-methyl-3-(3-phenyl-[1,2,4]triazolo*[3,4-b][1,3,4]thiadiazol-6-yl)-1H-indazole*

IR(KBr)*v*/cm^-1^:3064.12(Ar-Hstr),2937.11(methylC-Hstr),1599.45(C=Nstr), 1464.29(C=Cstr).^1^HNMR(400MHz,DMSO-d6):*δ*:8.4(d,1H,Ar-H),8.1(m,3H,Ar-H),8.0(m,1H,Ar-H),7.9(m,1H,Ar-H),7.7-7.6(m,1H,Ar-H),7.3-7.1(m,2H,Ar-H),3.6(s,3H,-CH_3_);13CNMR(DMSO-*d*6)*δ*:168.2,164.19,159.73,152.00, 147.45,132.26,127.27,126.64,123.40,121.70,118.93,114.48,113.62,110.66,45.38.; LCMS (MM:ES+APCI) 333.2(M+H)^+^


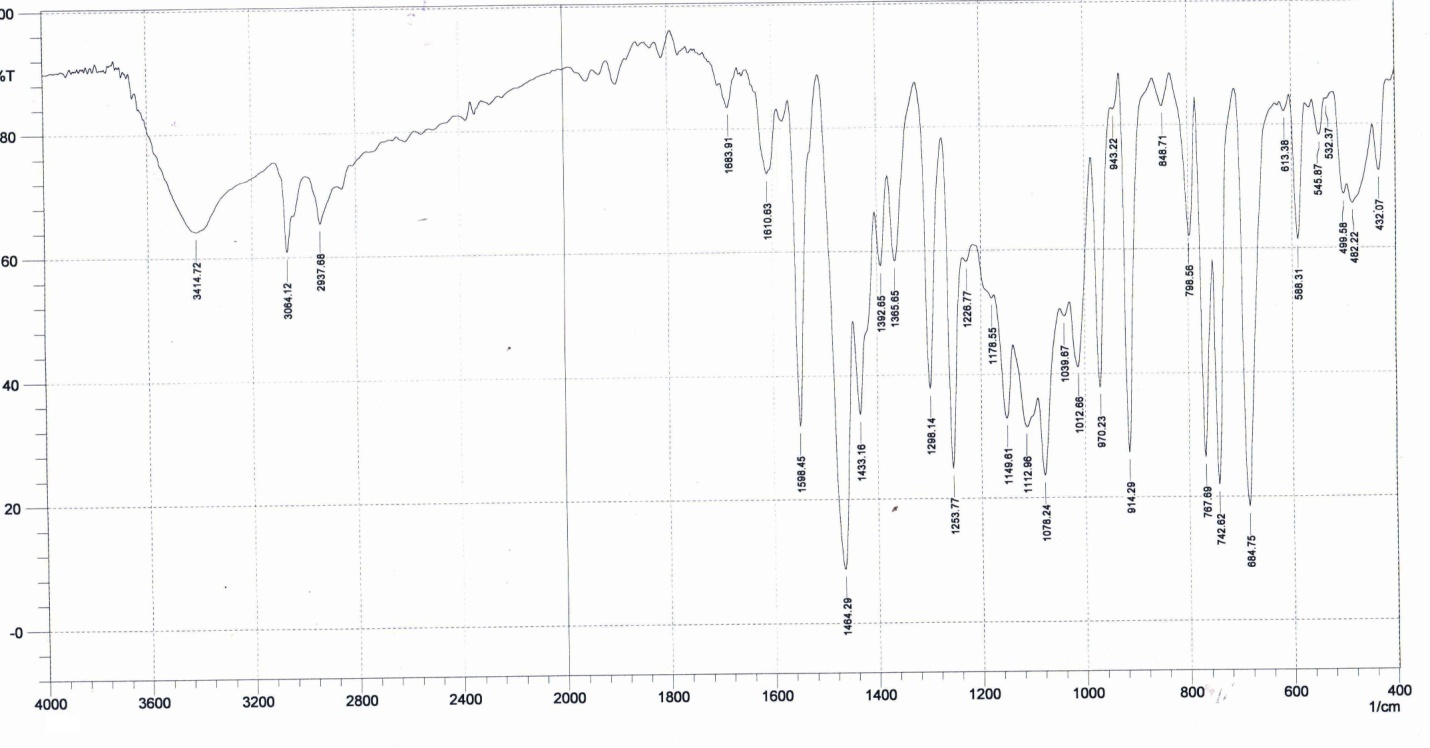


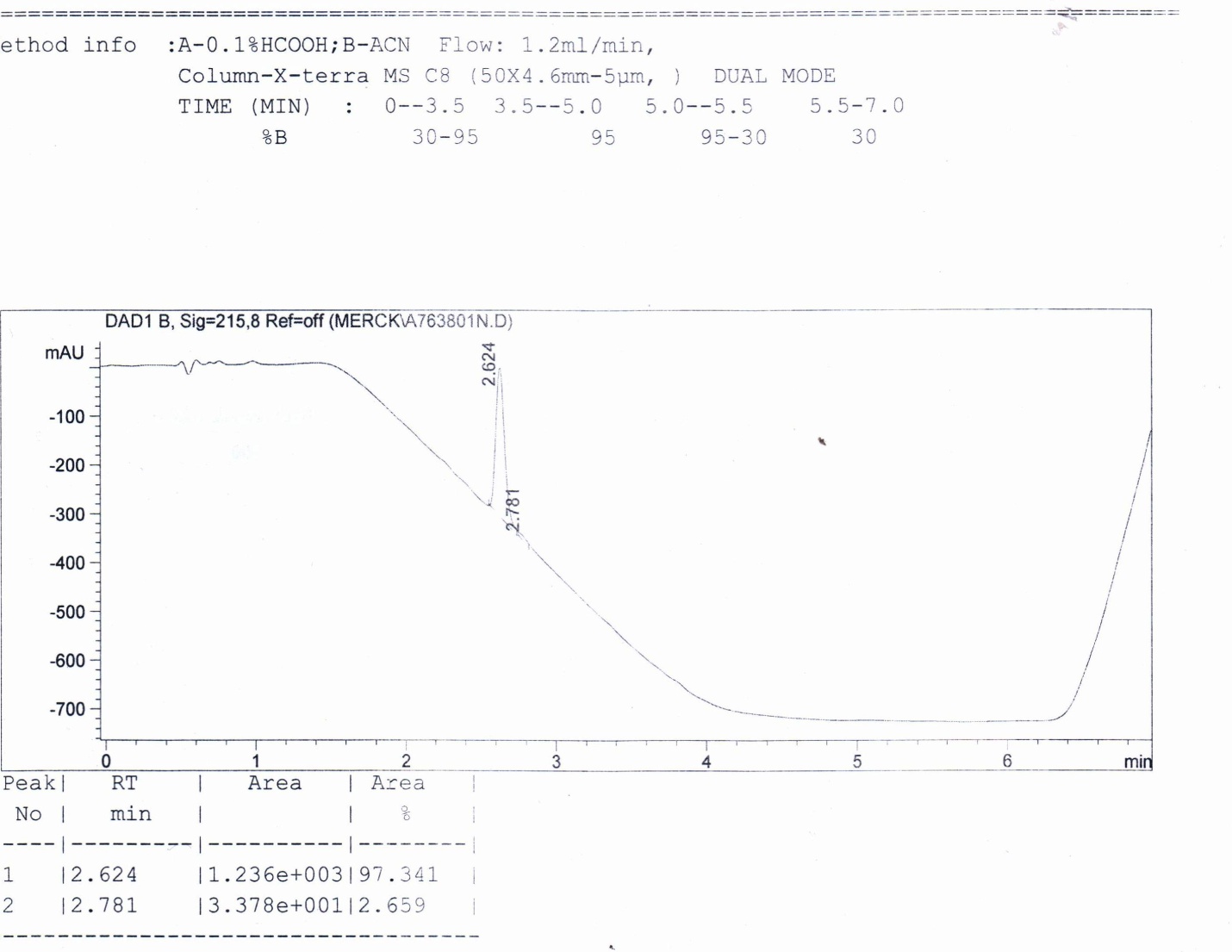


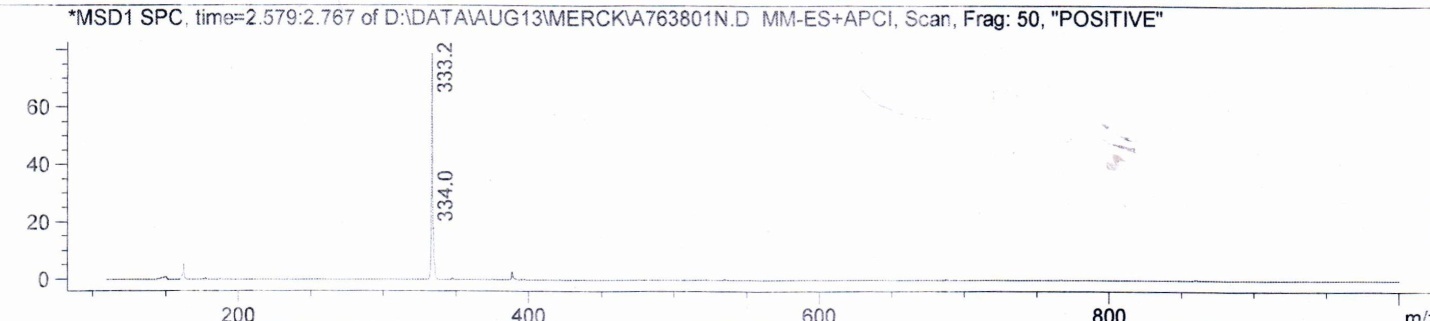


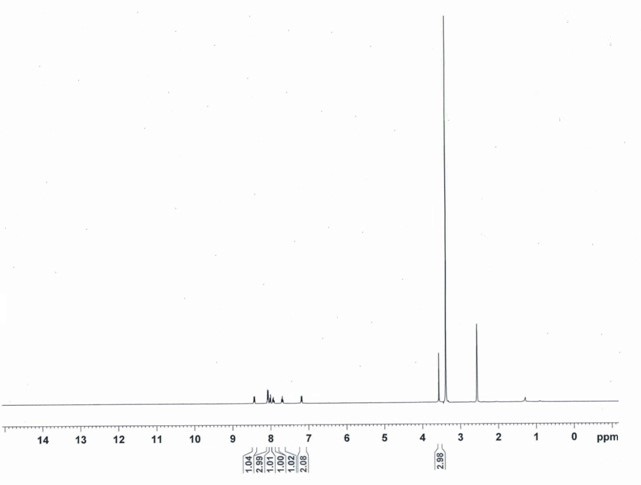


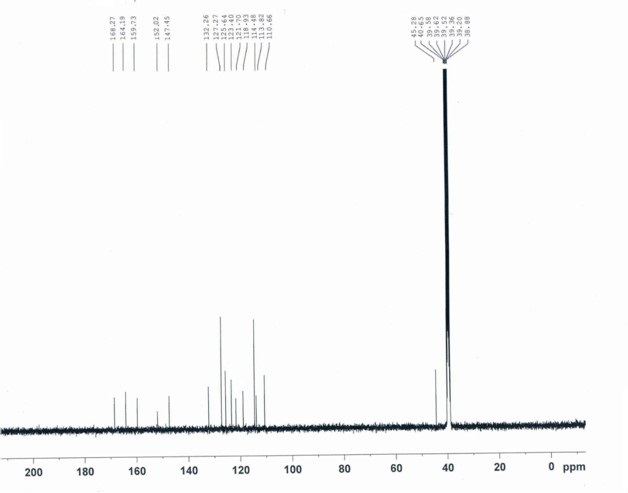


***(2e)***

6-(cylohexylmethyl)-3-phenyl-*[1,2,4]triazolo[3,4-b][1,3,4]thiadiazole*.

IR(KBr)*v*/cm^-1^:3035.68(ArC-Hstr),2966.38(methylC-Hstr),1605.45(C=Nstr), 1469.81(C=Cstr). ^1^HNMR(400MHz,DMSO-d6):8.2(d,2H,Ar-H),7.7-7.5(m,3H,Ar-H),2.9(d,2H,CH_2_),1.9-0.8(m,10H,Cyclohexyl),0.78(s,9H,-CH_3_);13CNMR (DMSO-*d*6) *δ*: 167.91, 164.82, 159.48,152.99,146.87,137.46,132.28,130.26,53.85, 46.13, 43.72, 28.63, 25.02, 21.13.; LCMS (MM:ES+APCI) 355.2(M+H)^+^


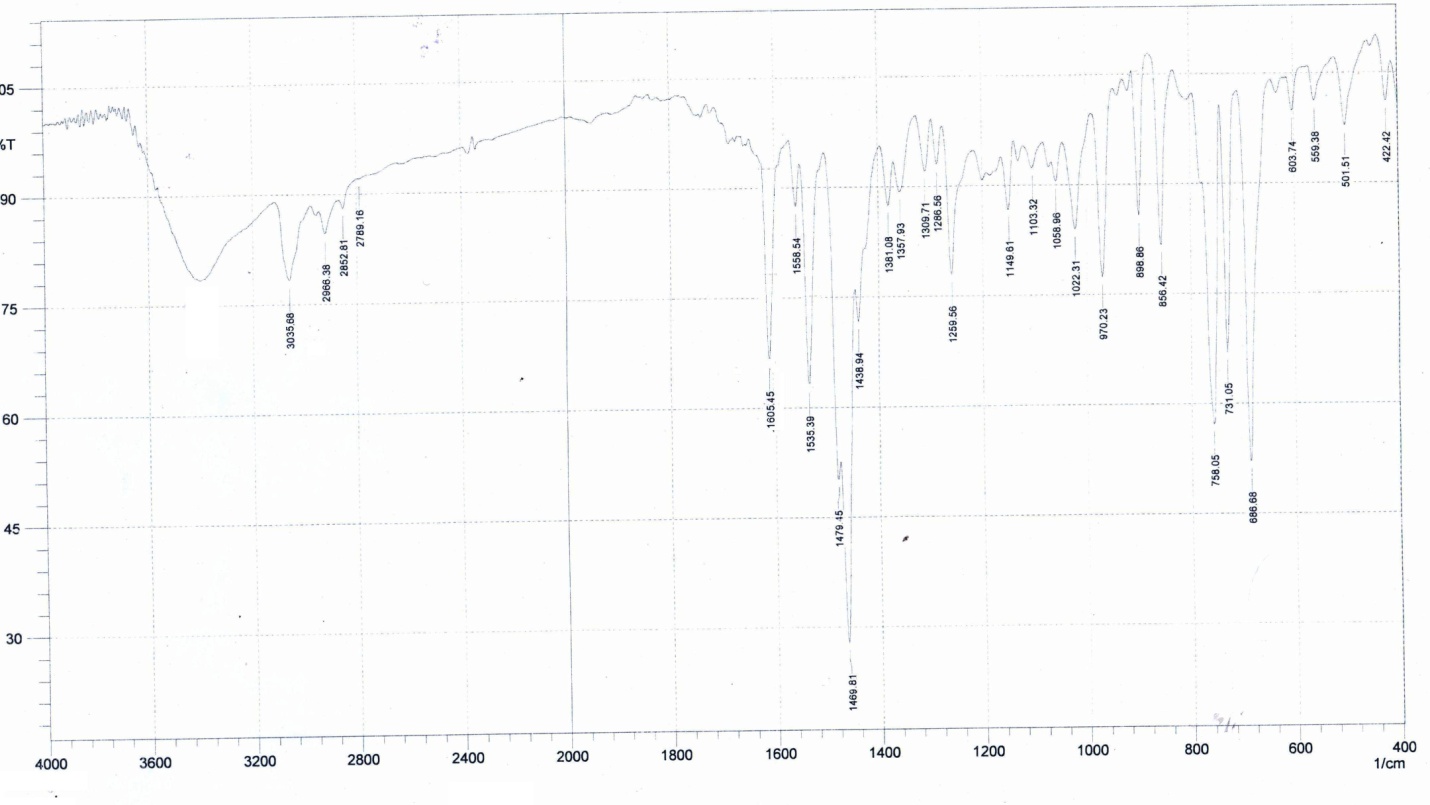


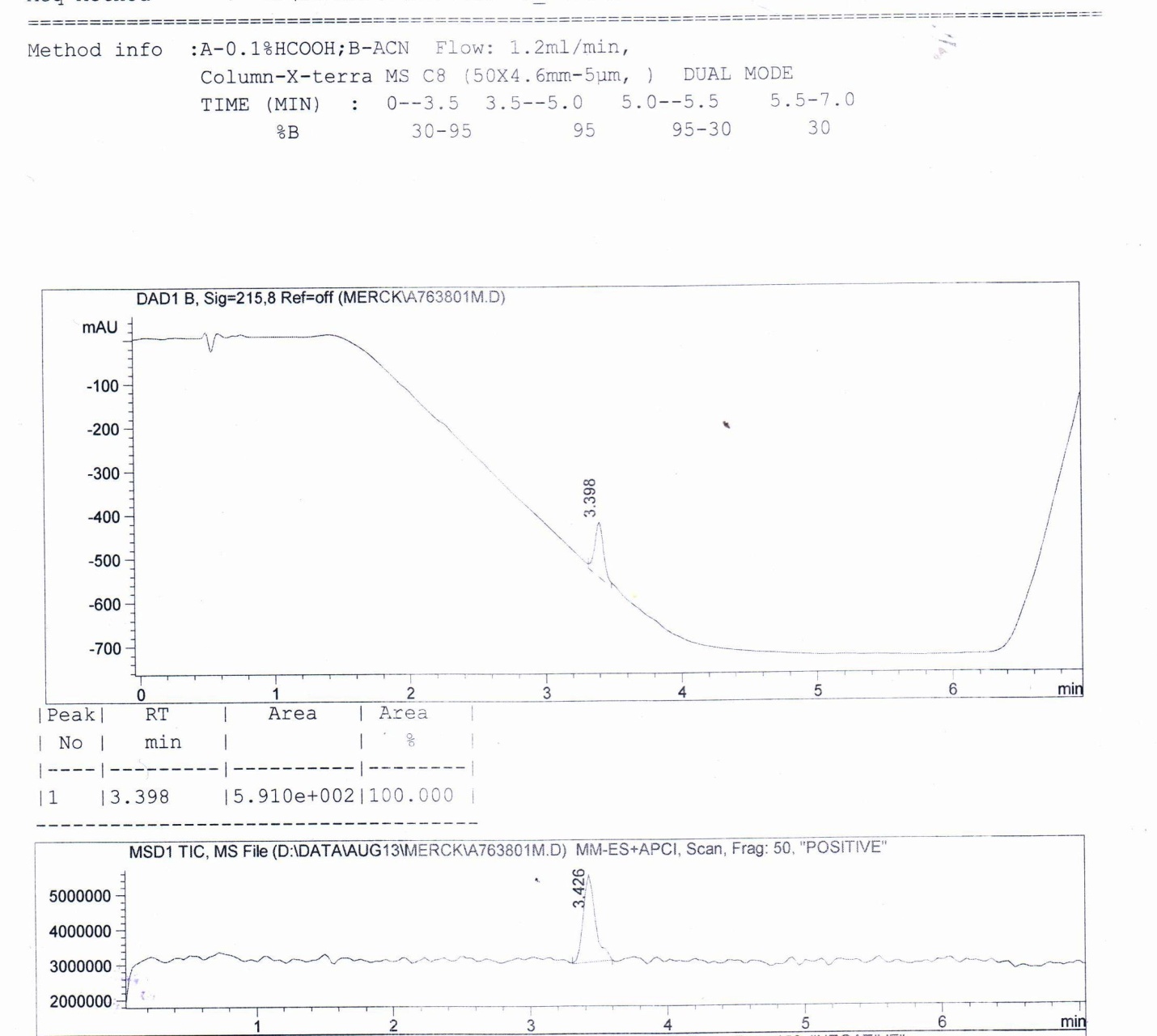


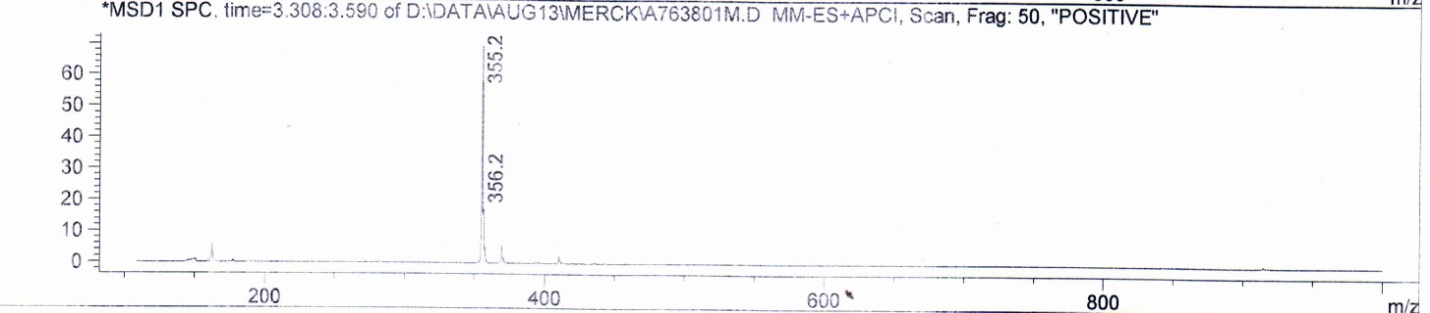


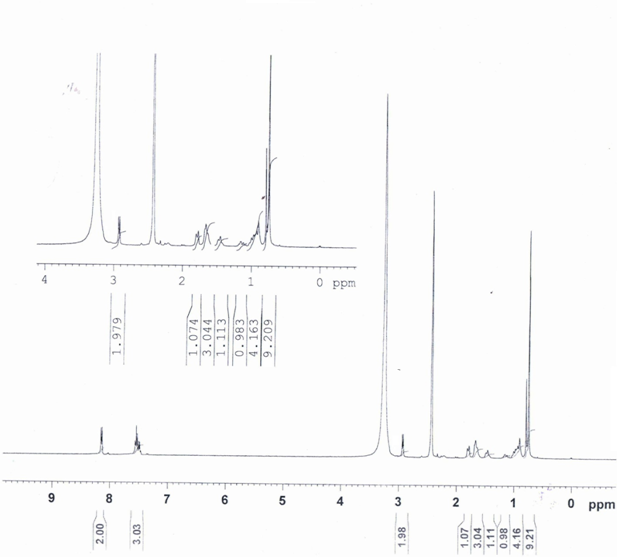


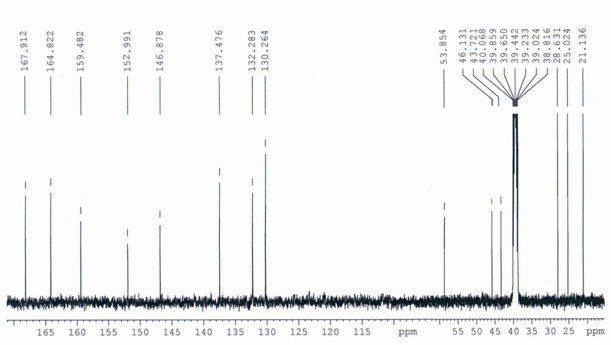


**(2f)**

6-(6-chloropyridin-2-yl)-3-phenyl-*[1,2,4]triazolo[3,4-b][1,3,4]thiadiazole***.**

IR(KBr)*v*/cm^-1^:3023.21(ArC-H),1604.83(C=Nstr),1469.81(C=Cstr),723.33(C-Clstr). ^1^HNMR(400MHz,DMSO-d6):*δ*:8.9(d,1H,Ar-H),8.8(d,1H,Ar-H), 8.2-8.3(m,2H,Ar-H),7.8-7.7(m,1H,Ar-H),7.5-7.6(m,3H,Ar-H):LCMS (MM:ES+APCI) 314.02(M+H)^+^


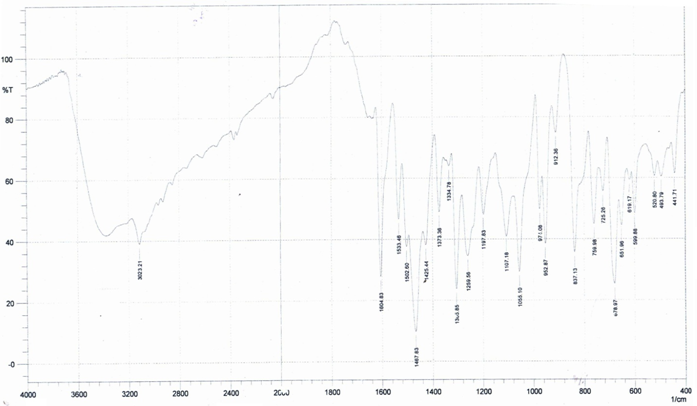


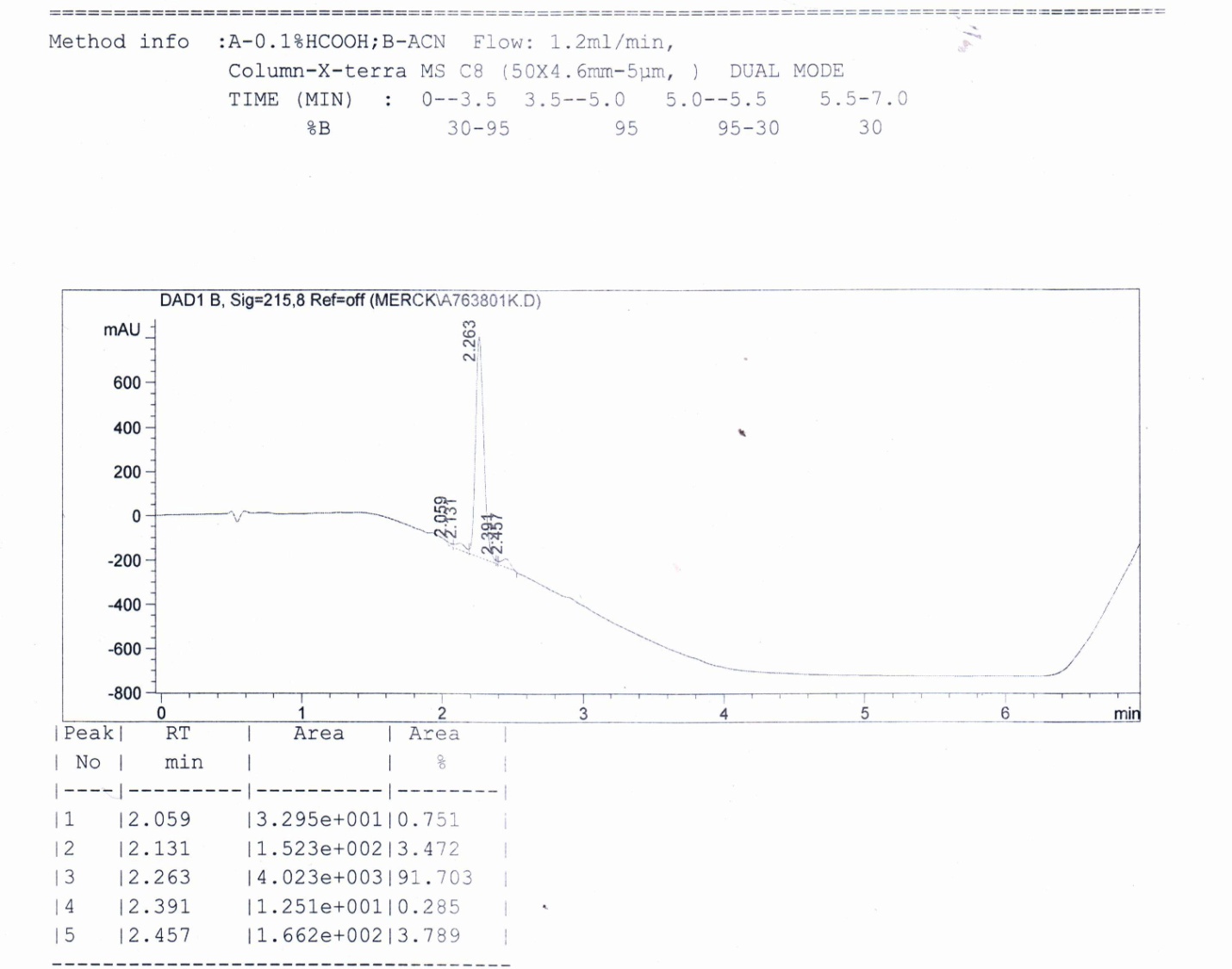


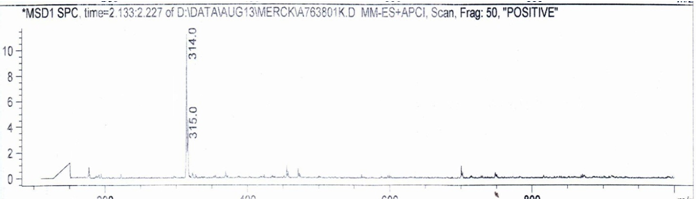


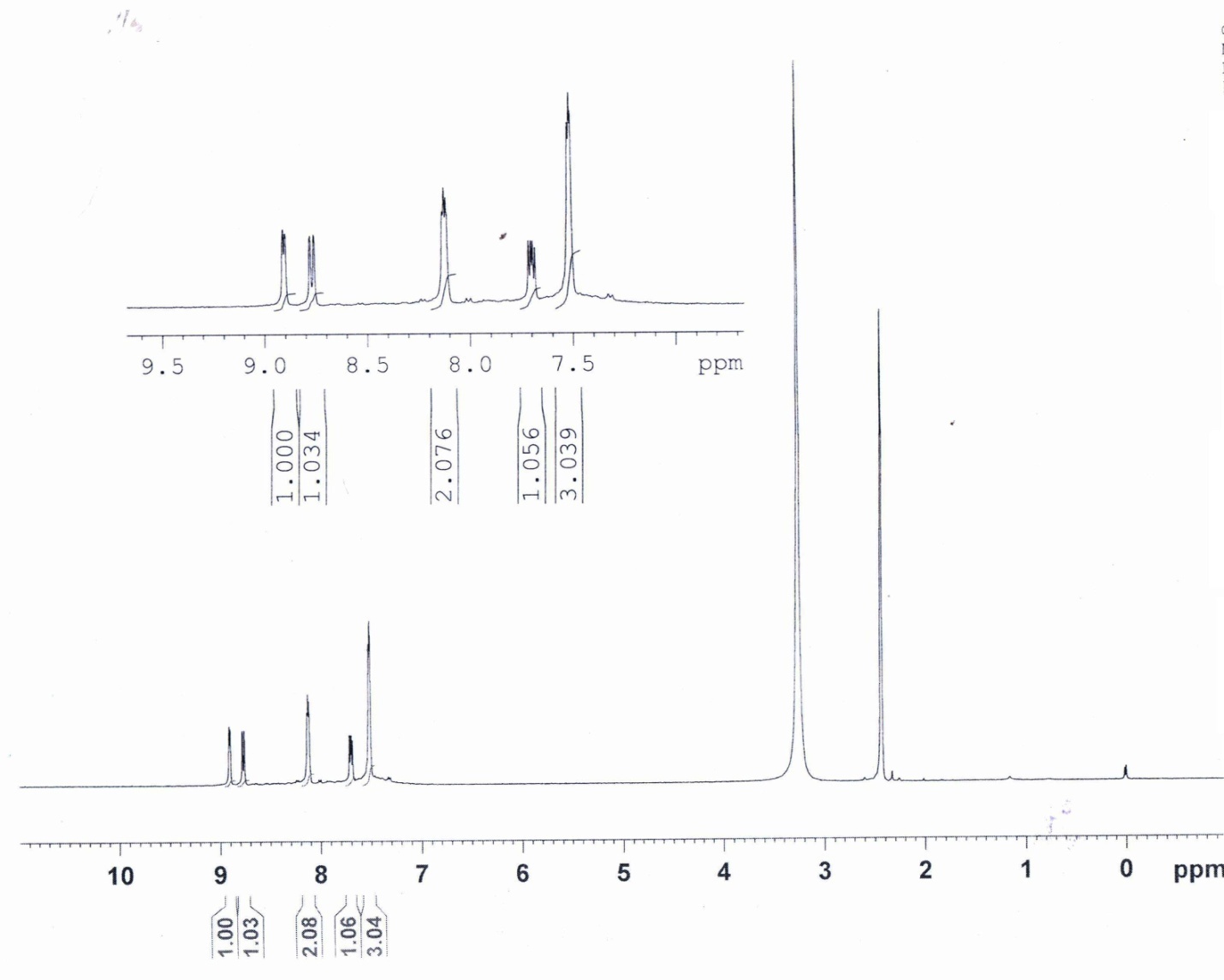


**(2g)**

(4-(3-phenyl-[*1,2,4]triazolo[3,4-b][1,3,4]thiadiazol-6-yl)pyridine-2-yl)methanamine*

IR(KBr)*v*/cm^-1^:3420.68(N-Hstr),3090.68(ArC-Hstr),1608.45(C=Nstr), 1471.44(C=Cstr). ^1^HNMR(400MHz,DMSO-d6): *δ*: 8.4 (d,1H,Ar-H), 8.2 (d,1H,Ar-H),7.7-7.5(m,3H,Ar-H),7.0-6.9(m,3H,Ar-H),4.6(m,2H,-CH_2_),2.8(s,2H,-NH_2_);13CNMR(DMSO-*d*6)δ:168.27,165.94,153.47,147.94, 145.12,136.38,134.36,131.13,128.78,126.02,125.58,124.84,119.40,109.63,72.33 ;LCMS (MM:ES+APCI) 309.0(M+H)^+^


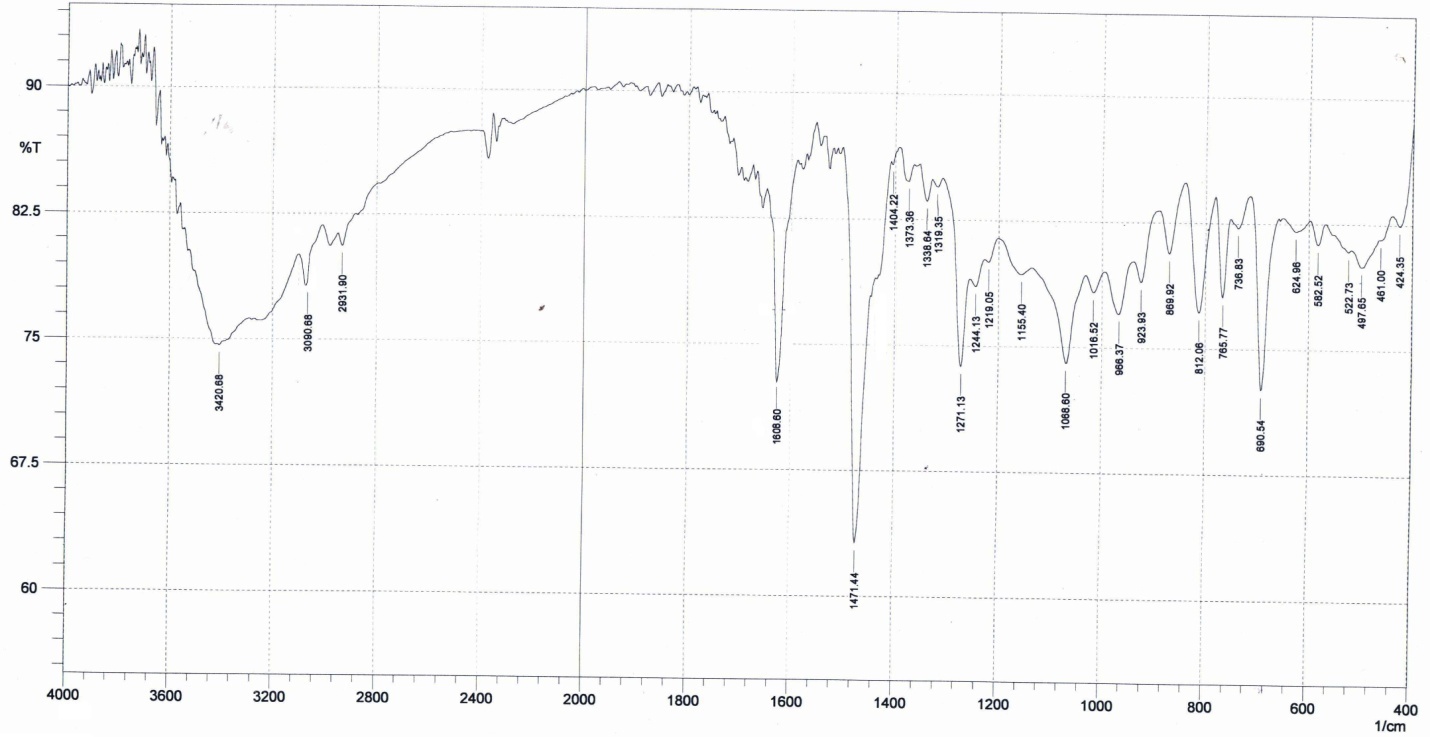


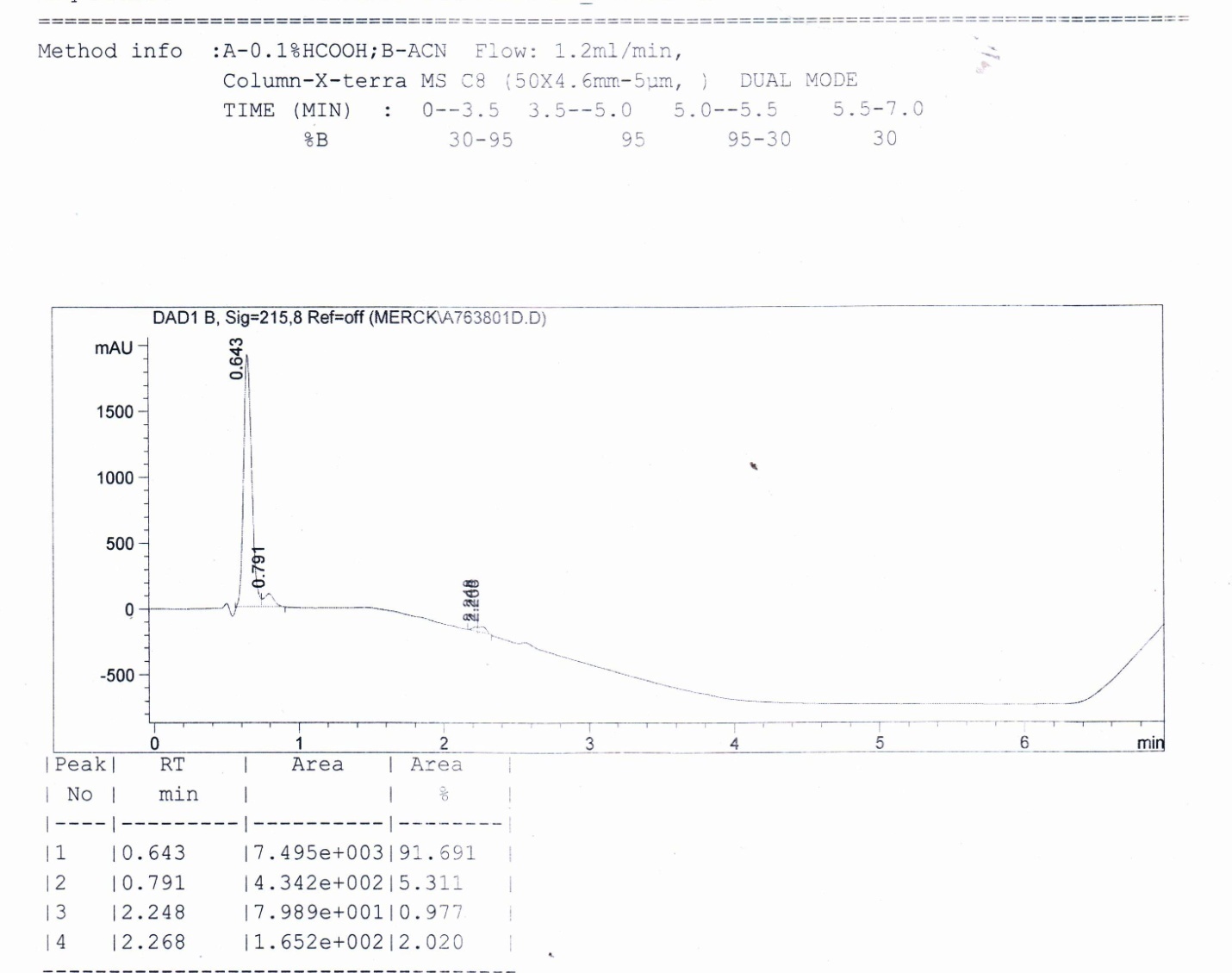


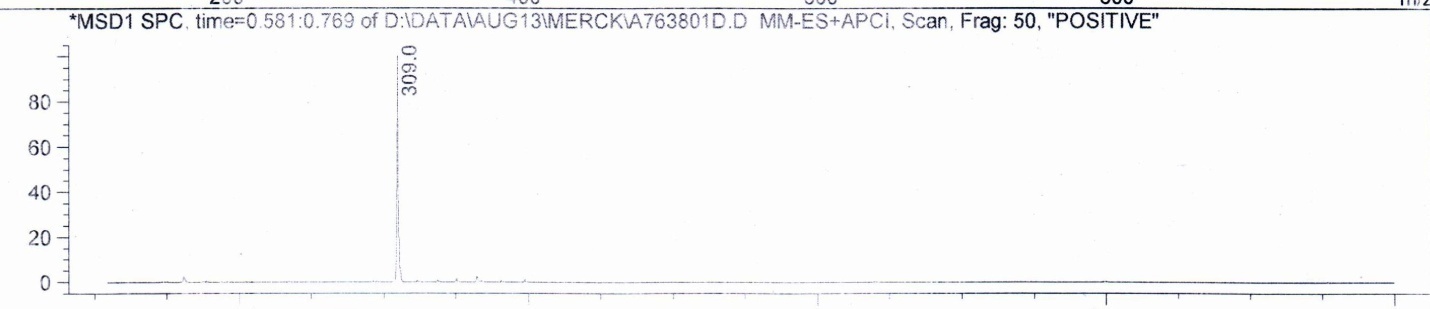


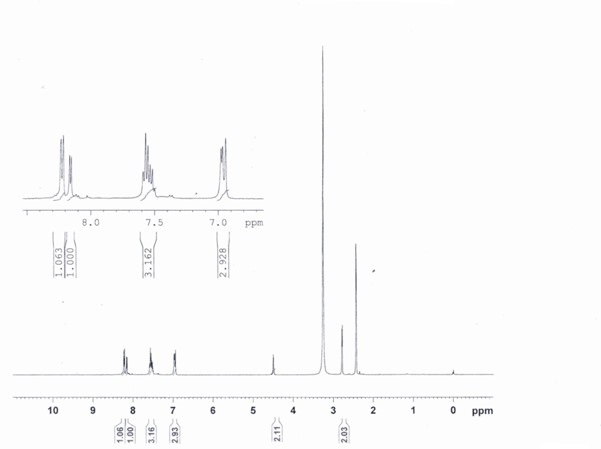


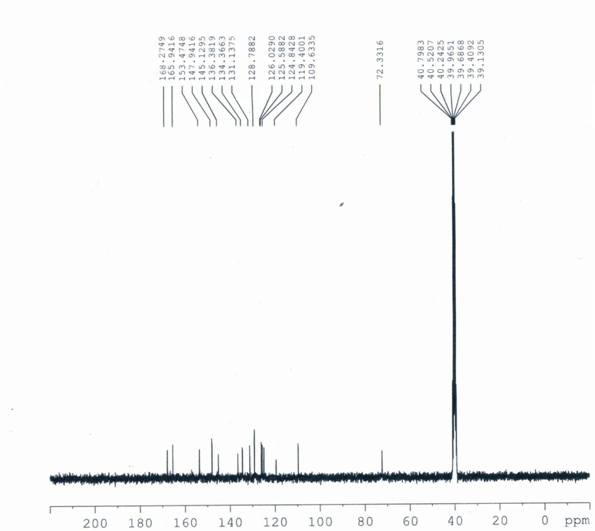


**(2h)**

6-(1-methyl-1H-pyrrol-2-yl)-3-phenyl-*[1,2,4]triazolo[3,4-b][1,3,4]thiadiazole*

IR(KBr)*v*/cm^-1^:3029.20(ArC-Hstr),2902.25(methylC-Hstr),1606.76(C=Nstr), 1469.54(C=Cstr):^1^HNMR(400MHz,DMSO-d6): *δ*: 8.4 (d,1H,Ar-H), 8.0 (d,1H,Ar-H),7.9(m,1H,Ar-H),7.8(d,1H,Ar-H)7.7-7.6(m,1H,Ar-H),6.9(s,1H,Pyrole-CH),6.1-6.00(m,2H,Pyrole-CH), 3.5(s, 3H,-CH_3_); 13C NMR (DMSO-*d*6) *δ*: 168.2, 161.9, 159.9,155.6,145.4,136.5,122.9, 120.2, 116.6, 112.5,37.2 ; LCMS (MM:ES+APCI) 282.0(M+H)^+^


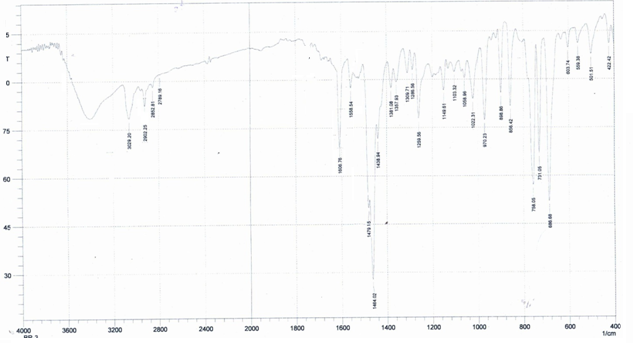


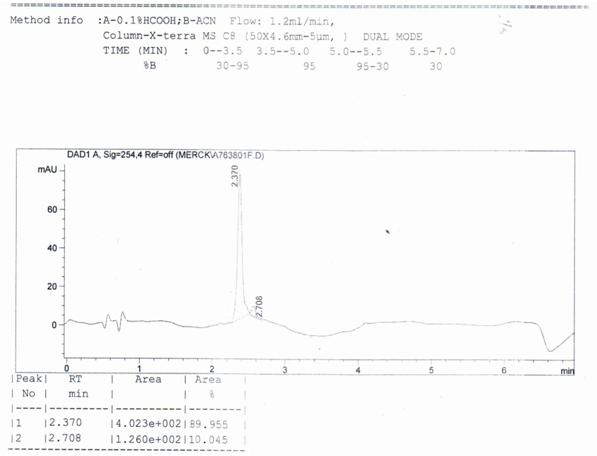


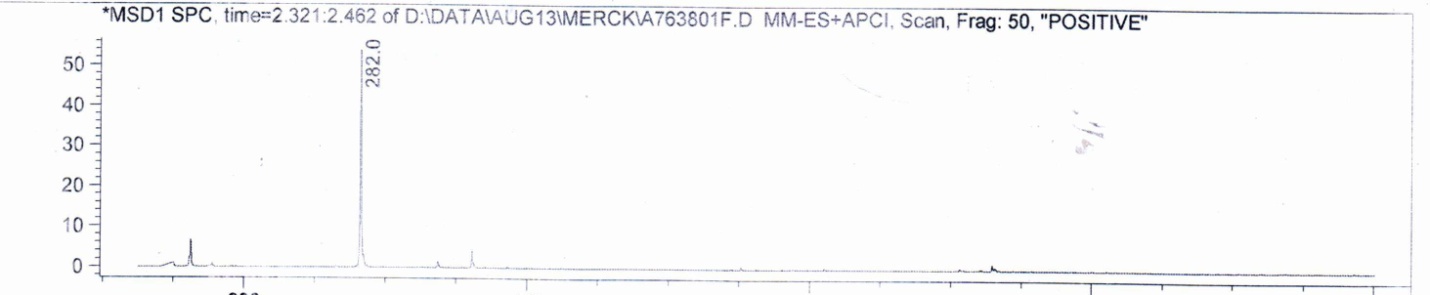


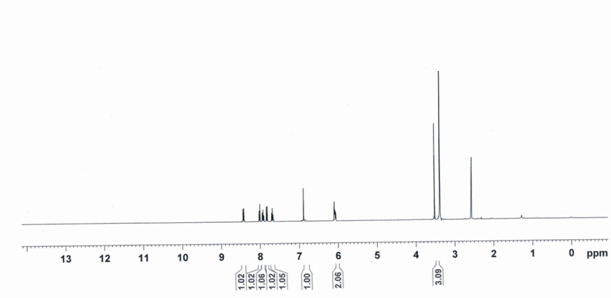


**(2i)**

6-(benzo[d][1,3]dioxol-5-yl)-3-phenyl-*[1,2,4]triazolo[3,4-b][1,3,4]thiadiazole*

IR(KBr)*v*/cm^-1^:3030.55(ArC-Hstr),1585.43(C=Nstr),1471.88(C=Cstr). ^1^HNMR(400MHz,DMSO-d6):*δ*:8.3(d,2H,Ar-H),7.6-7.4(m,5H,Ar-H),7.1(d,1H,Ar-H),6.1(s,2H,-CH_2_),LCMS(MM:ES+APCI)323.0(M+H)^+^.


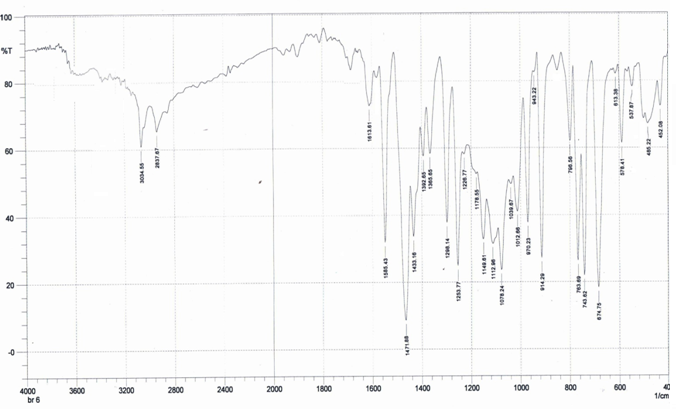


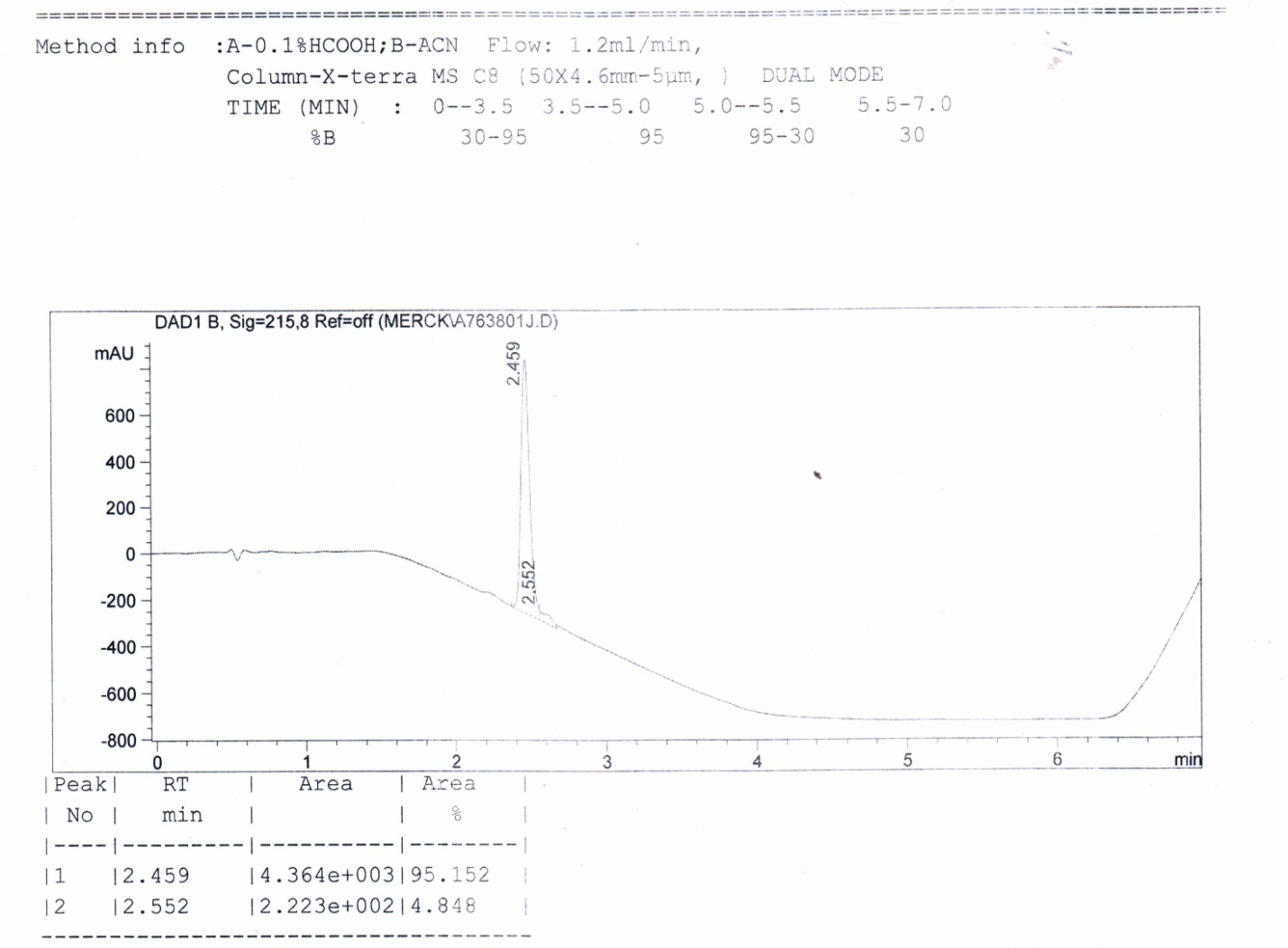


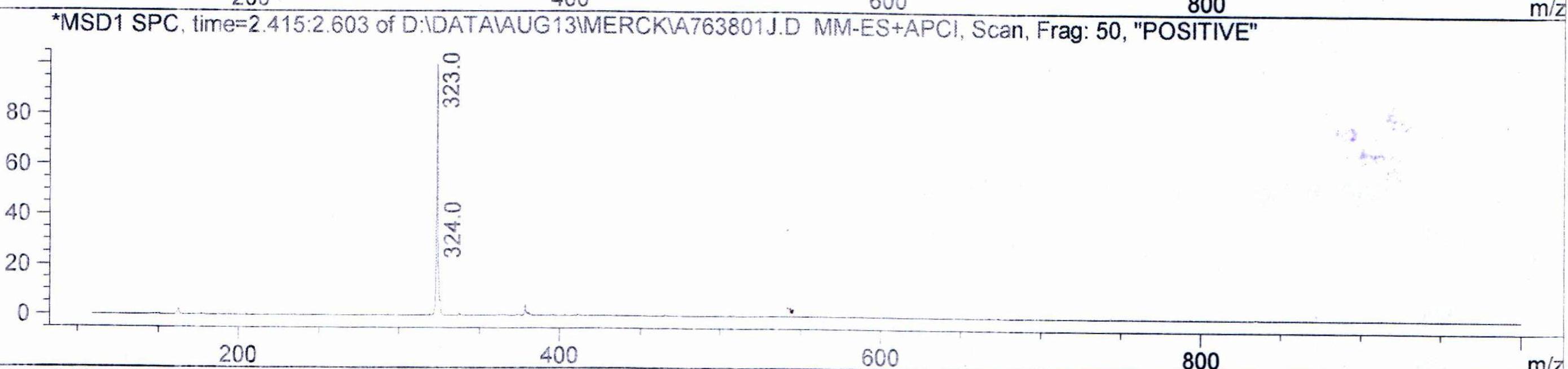


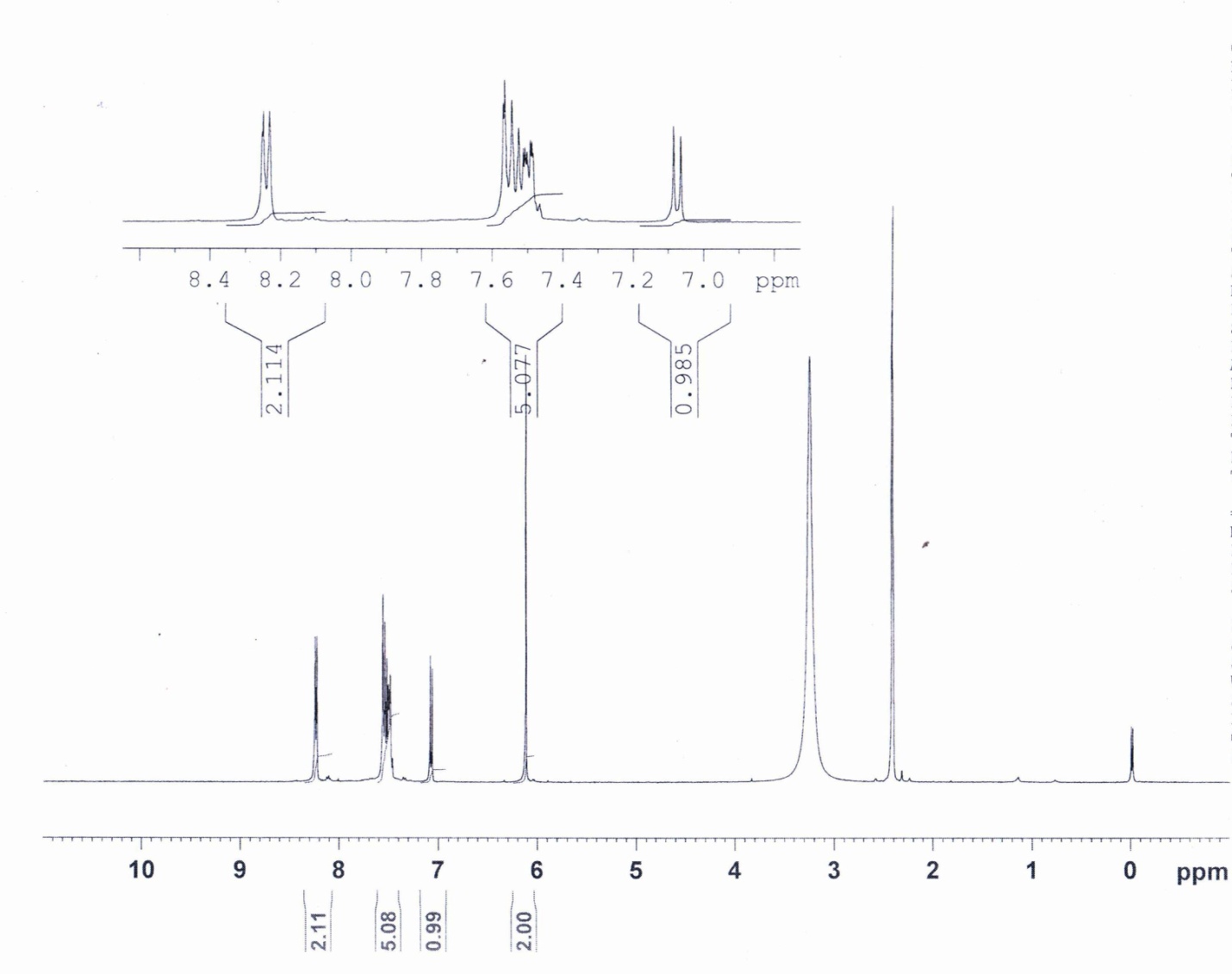


**(2j)**

3-(trifluoromethyl)-2-methyl-N-(2-(3-phenyl-*[1,2,4]triazolo[3,4-b][1,3,4]thiadiazol-6-yl)benzenamine*

IR(KBr)*v*/cm^-1^:3090.68(ArC-Hstr),1605.45(C=Nstr),1467.88(C=C). ^1^HNMR(400MHz,DMSO-d6):*δ*:9(s1H,Ar-H),8.3(d,1H,Ar-H),8.2(m,2H,Ar-H),8.1(d,1H,Ar-H),7.8(dd,1,H,Ar-H),7.5(m,3H,Ar-H),7.4(d,2H,Ar-H),7.3(m,1H,Ar-H),7.0(m,1H,Ar-H),2.2(s,3H,-CH_3_).; LCMS (MM:ES+APCI) 452.0(M+H)^+^


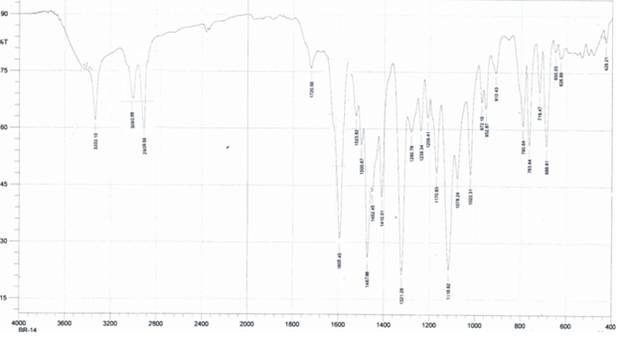


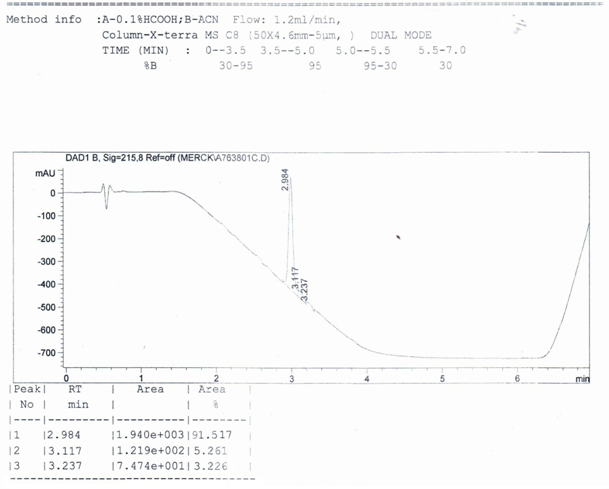


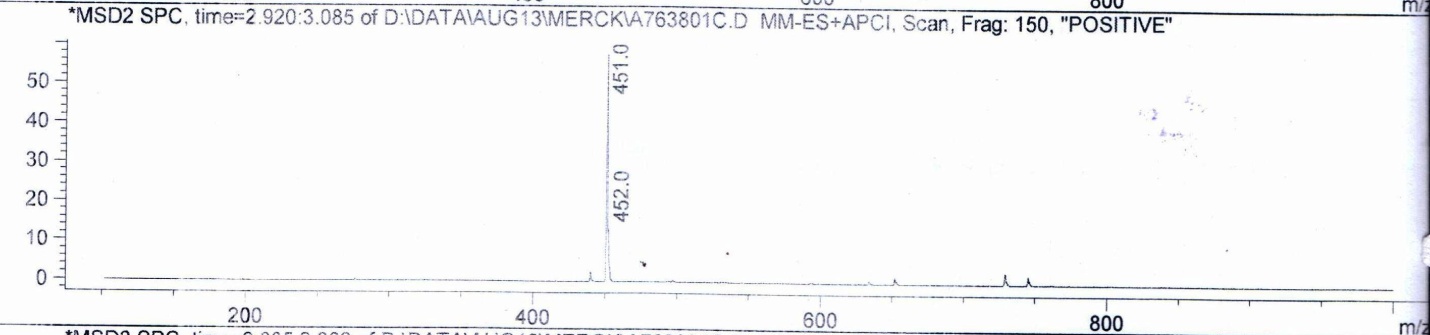


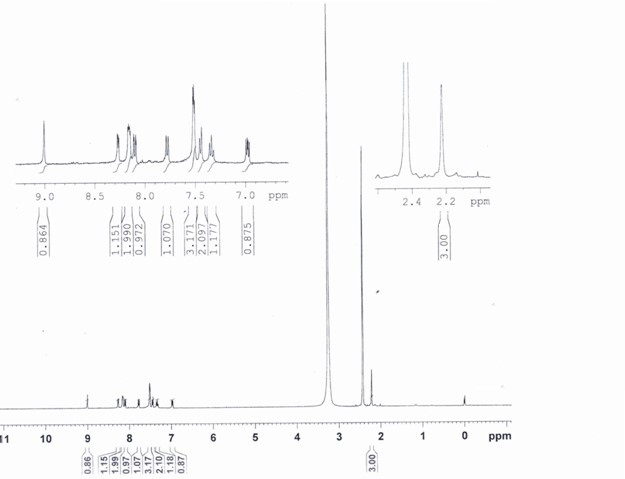


**(2k)**

6-(2-benzylphenyl)-3-phenyl-*[1,2,4]triazolo[3,4-b][1,3,4]thiadiazole*.

IR(KBr)*v*/cm^-1^:3034.78 (ArC-Hstr), 2858.18(methylC-Hstr),1599.45(C=Nstr), 1467.88(C=Cstr).^1^HNMR(400MHz,DMSO-d6): *δ*: 8.0(d,2H,Ar-H), 7.7(d,1H,Ar-H), 7.6(m,1H,Ar-H),7.5-7.3(m, 6H,Ar-H), 7.2(m, 2H,Ar-H),7.0(d,2H,Ar-H),4.3(s,2H,-CH_2_) ; 13C NMR (DMSO-*d*6) *δ*: 168.89, 164.19, 159.73,152.02, 147.45, 142.43, 138.44,137.46,132.26,127.27,125.64,123.40,121.70,55.23,;LCMS (MM:ES+APCI) 369.3(M+H).^+^


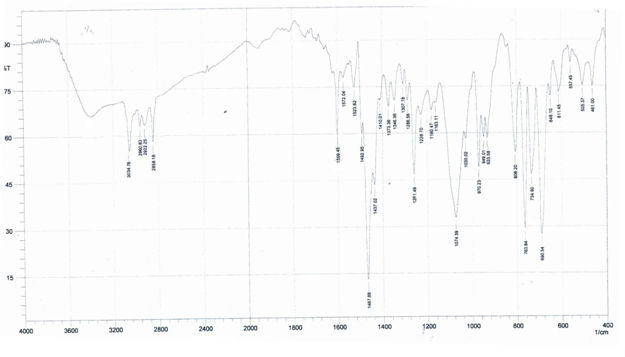


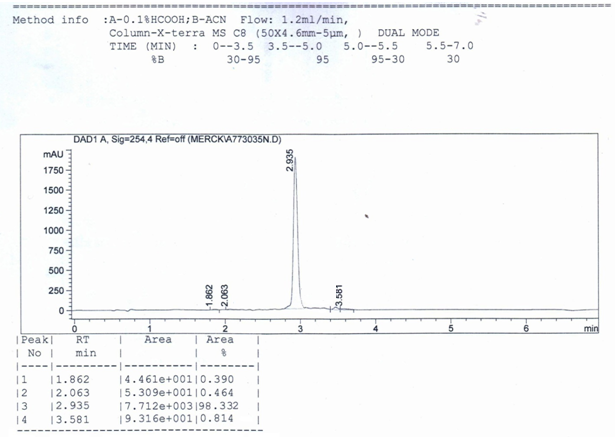


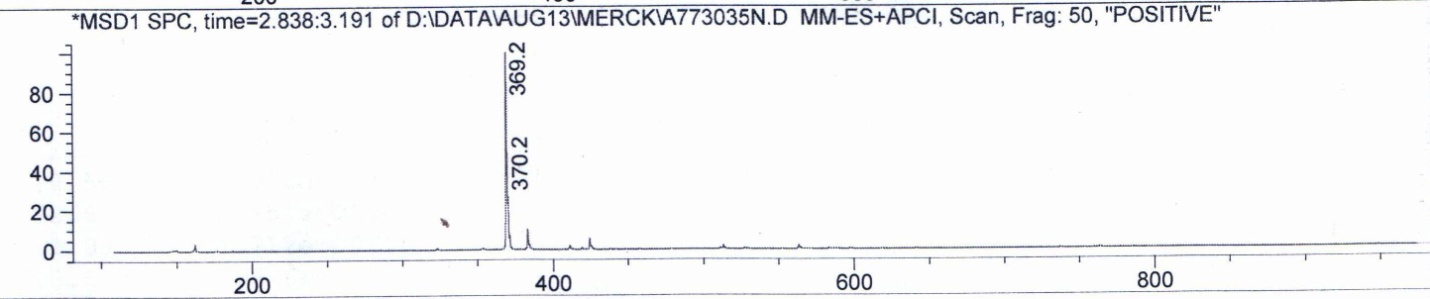


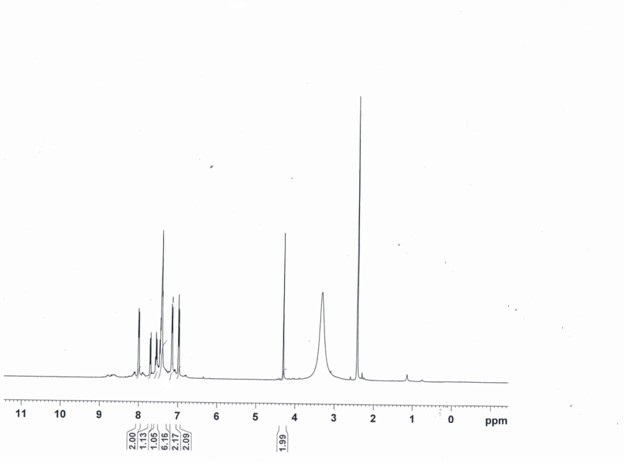


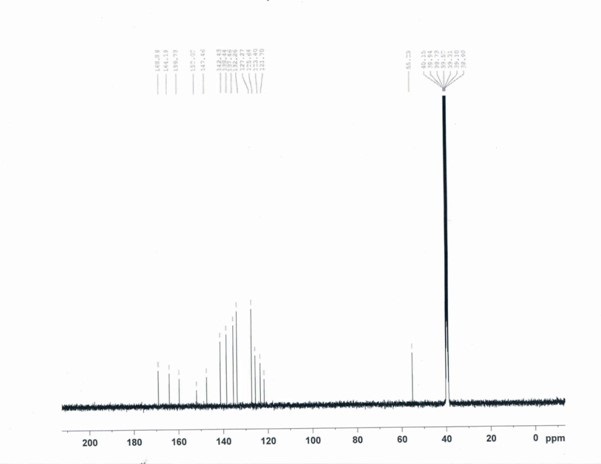


**(2l)**

3-(3-phenyl-*[1,2,4]triazolo[3,4-b][1,3,4]thiadiazol-6yl)-1-p-tolylpropan-1-one.*

IR(KBr)*v*/cm^-1^:3068.85(ArC-Hstr),2931.90(methyC-Hstr),1662.69(=COstr) 1608.69(C=Nstr),1462.09(C=Cstr). ^1^HNMR(400MHz,DMSO-d6):*δ*:8.3 (m,1H,Ar-H),8.2(m,1H,Ar-H),8.1(m,2H,Ar-H),7.9-7.7(m,3H,Ar-H),7.5(m,2H,Ar-H), 3.1(m,2H,-CH_2_),2.8(m,2H,-CH_2_),2.3(s,3H,-CH_3_) ; 13C NMR (DMSO-*d*6) *δ*: 179.35,167.81,164.13,158.14,152.17,149.20,138.81,135.10,132.39,130.13,127.60,125.99,124.24,36.13,29.03,22.00.; LCMS (MM:ES+APCI) 349.2(M+H)^+^


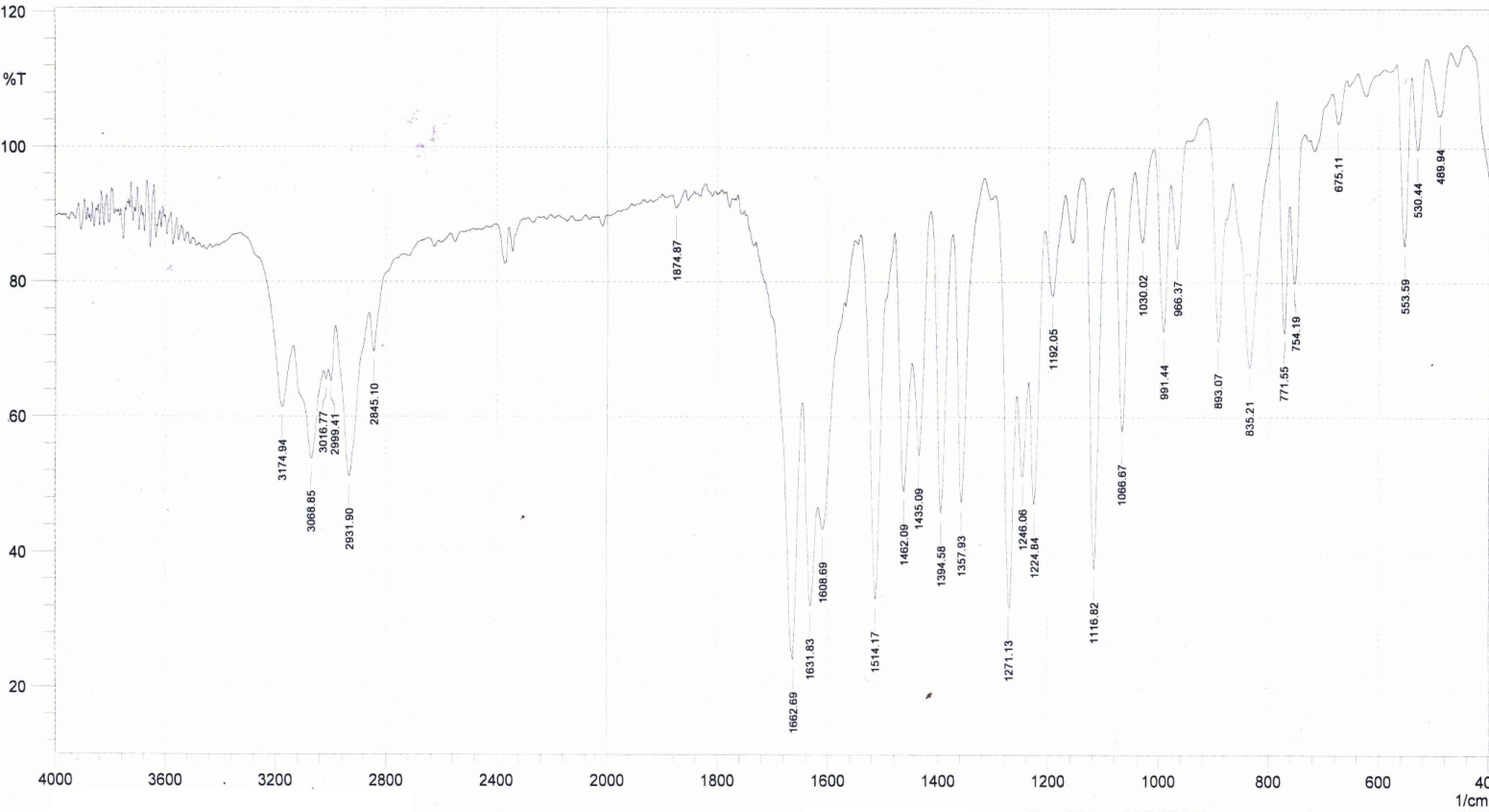


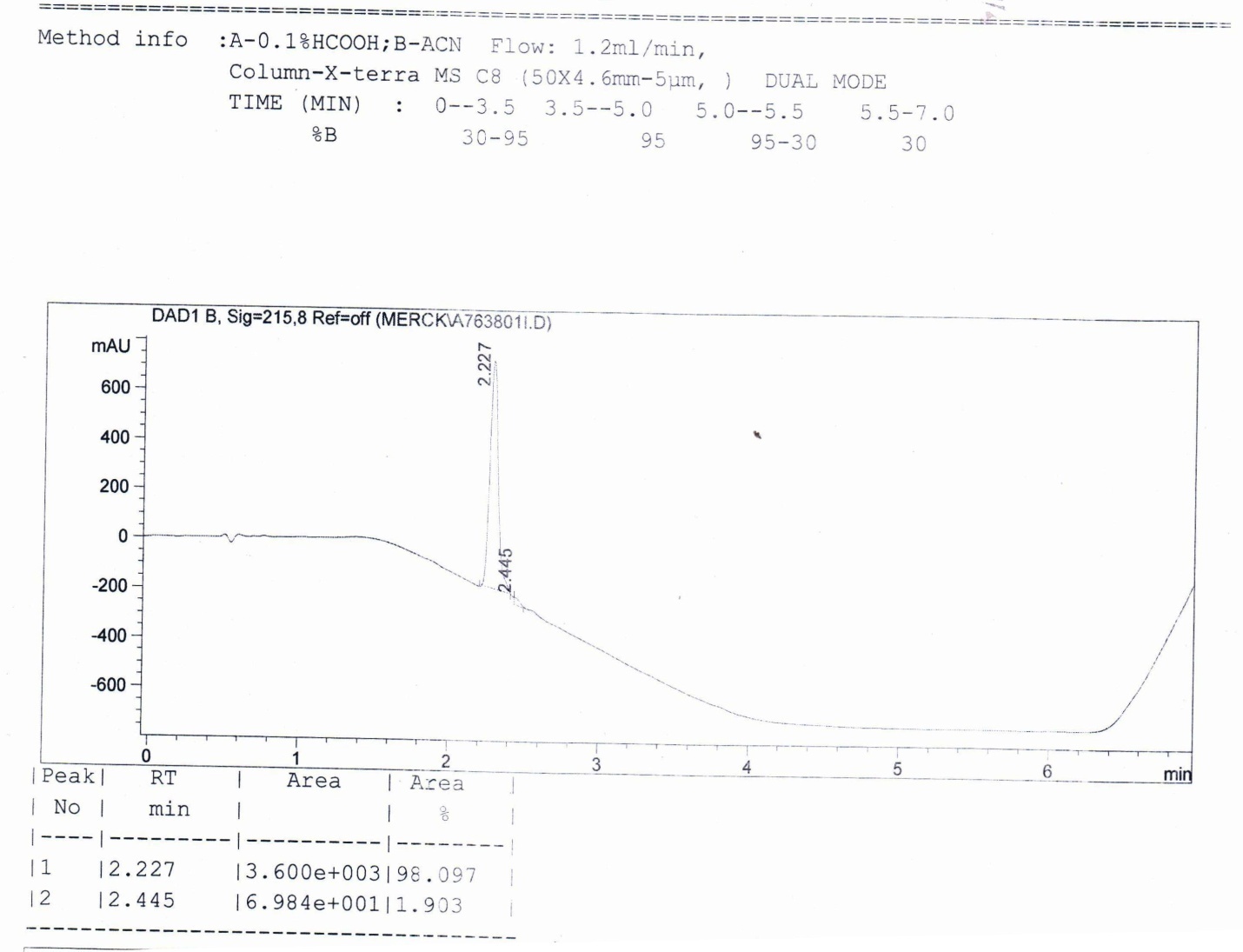


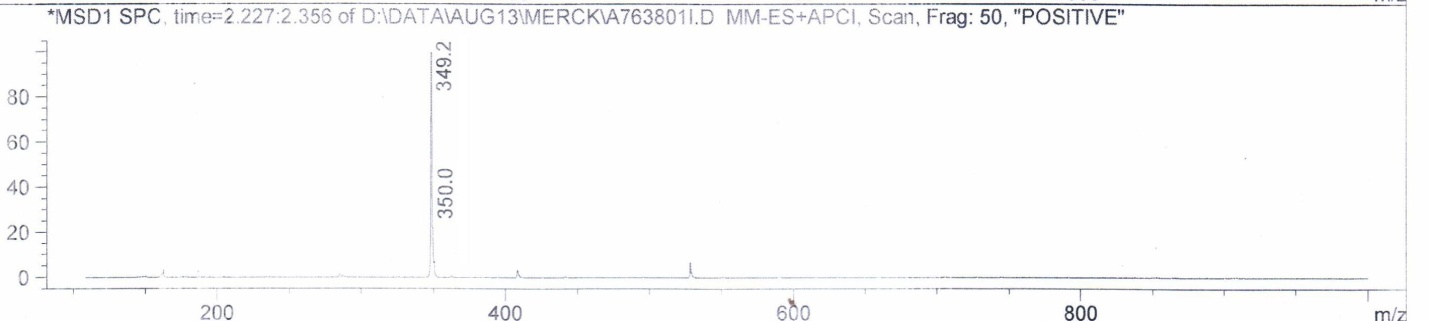


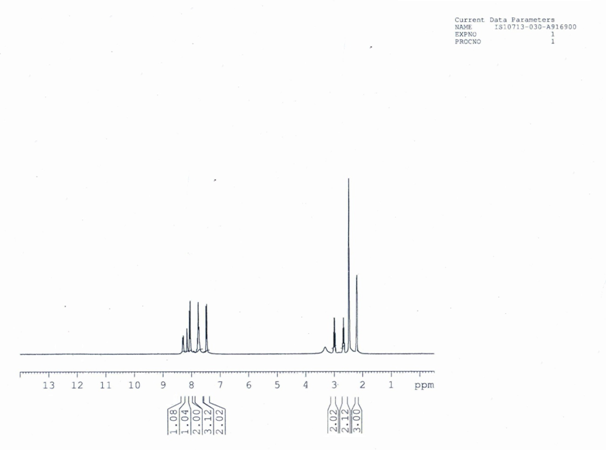


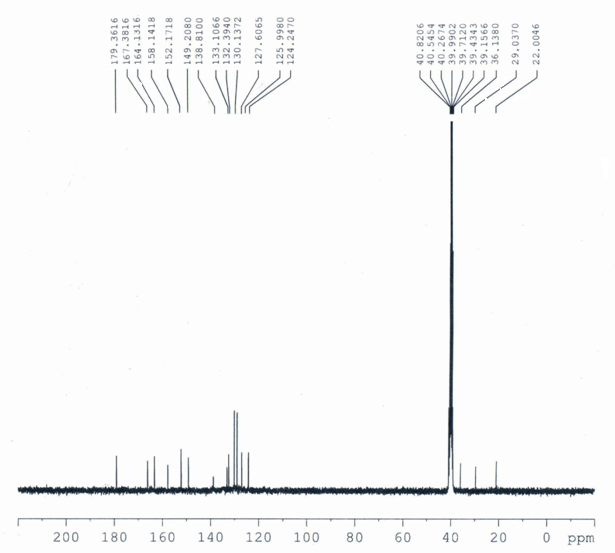


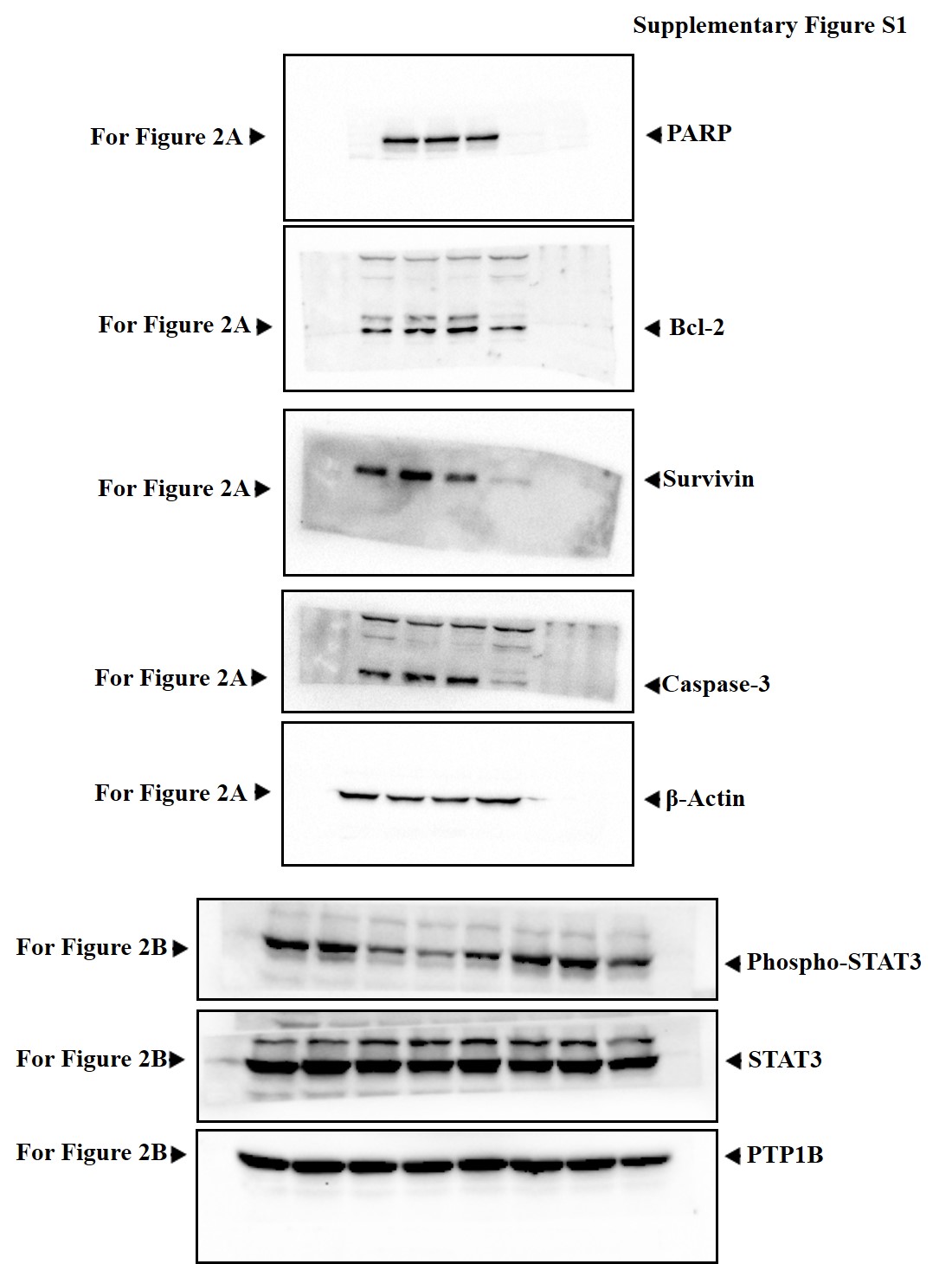

Supplement: Supplementary Information [file srep14195-s1.doc]
